# Silyl Ketene Acetals/B(C_6_F_5_)_3_ Lewis Pair-Catalyzed Living Group Transfer Polymerization of Renewable Cyclic Acrylic Monomers

**DOI:** 10.3390/molecules23030665

**Published:** 2018-03-15

**Authors:** Lu Hu, Wuchao Zhao, Jianghua He, Yuetao Zhang

**Affiliations:** State Key Laboratory of Supramolecular Structure and Materials, College of Chemistry, Jilin University, Changchun 130012, China; hulu14@mails.jlu.edu.cn (L.H.); zhaowc16@mails.jlu.edu.cn (W.Z.)

**Keywords:** Frustrated Lewis Pair, group transfer polymerization, silyl ketene acetal, B(C_6_F_5_)_3_

## Abstract

This work reveals the silyl ketene acetal (SKA)/B(C_6_F_5_)_3_ Lewis pair-catalyzed room-temperature group transfer polymerization (GTP) of polar acrylic monomers, including methyl linear methacrylate (MMA), and the biorenewable cyclic monomers γ-methyl-α-methylene-γ-butyrolactone (MMBL) and α-methylene-γ-butyrolactone (MBL) as well. The in situ NMR monitored reaction of SKA with B(C_6_F_5_)_3_ indicated the formation of Frustrated Lewis Pairs (FLPs), although it is sluggish for MMA polymerization, such a FLP system exhibits highly activity and living GTP of MMBL and MBL. Detailed investigations, including the characterization of key reaction intermediates, polymerization kinetics and polymer structures have led to a polymerization mechanism, in which the polymerization is initiated with an intermolecular Michael addition of the ester enolate group of SKA to the vinyl group of B(C_6_F_5_)_3_-activated monomer, while the silyl group is transferred to the carbonyl group of the B(C_6_F_5_)_3_-activated monomer to generate the single-monomer-addition species or the active propagating species; the coordinated B(C_6_F_5_)_3_ is released to the incoming monomer, followed by repeated intermolecular Michael additions in the subsequent propagation cycle. Such neutral SKA analogues are the real active species for the polymerization and are retained in the whole process as confirmed by experimental data and the chain-end analysis by matrix-assisted laser desorption/ionization time of flight mass spectroscopy (MALDI-TOF MS). Moreover, using this method, we have successfully synthesized well-defined PMMBL-*b*-PMBL, PMMBL-*b*-PMBL-*b*-PMMBL and random copolymers with the predicated molecular weights (*M*_n_) and narrow molecular weight distribution (MWD).

## 1. Introduction

Lewis pair (LP) polymerization has emerged and attracted intense investigations of the cooperative (or synergistic) catalytic effects of Lewis acid (LA) and Lewis base (LB) pairs on the polymerization of conjugated polar alkenes [1,2,3,4,5,6,7,8], since the seminal works on Frustrated Lewis Pairs (FLPs) by Stephan and Erker in small molecule chemistry [9,10,11,12]. For instance, LPs based on strongly acidic, bulky E(C_6_F_5_)_3_ (E = B, Al) LAs and bulky LBs including phosphines and *N*-heterocyclic carbenes (NHCs) have been employed to initiate rapid addition polymerization of conjugated polar vinyl monomers such as methyl methacrylate (MMA), cyclic and the naturally renewable monomers α-methylene-γ-butyrolactone (MBL), and γ-methyl-α-methylene-γ-butyrolactone (MMBL) [13,14,15] and monomers bearing the C=C–C=N functionality such as 2-vinylpyridine and 2-isopropenyl-2-oxazoline as well [16,17]. In such polymerizations, the cooperativity of the LA and LB sites of Lewis pairs is essential to achieve an effective polymerization system, which was demonstrated by the borane/phosphine LPs that showed that interacting LPs, and even classical Lewis adducts (CLAs), can be highly active for the polymerization [18]. This was further nicely demonstrated by Rieger and co-workers, showing the high activity and high degree of control over the polymerization of Michael-type and extended Michael monomer systems by the highly interacting organoaluminum and phosphine LPs [19]. Extending beyond the commonly employed NHC and phosphine LBs, in 2014, Lu and co-workers first reported the *N*-heterocyclic olefin-based LPs for the polymerization of acrylamides, (meth)acrylates and disymmetric divinyl polar monomers as well [20,21]. Although FLPs or CLAs exhibited high activity for polymerization of conjugated polar alkenes, the application of such polymerization is hampered by both low initiation efficiencies and chain-termination side reactions [18,21], evidenced by the much higher observed *M*_n_ than the calculated *M*_n_ and broad MWD of the resulting polymers (high *Ð* values), thus giving rise to low initiation efficiencies (I*) and rendering the inability to produce well-defined block copolymers. Based on the above facts, it is really difficult to achieve LP-catalyzed living/controlled polymerization of polar acrylic monomers. More recently, Taton and co-workers reported the direct employment of organic LPs based on phosphine and Me_3_SiNTf_2_ for living polymerization of MMA, which proceeds through a similar mechanism compared to the group transfer polymerization (GTP) of MMA [22]. 

GTP is a successful, commercialized strategy for living polymerization of polar vinyl monomers proceeding through Mukaiyama-Michael reactions [23,24,25]. Such GTP process could be catalyzed by nucleophilic anionic bases, such as SiMe_3_F_2_^−^ [23,26,27], HF_2_^−^ [23,26,27,28], F^−^ [27,28,29], CN^−^ [23,27,28,29], N3^−^ [23,28], oxyanions[30,31], hydrogen bioxyanions [30,31,32]. Recently, neutral Lewis bases such as NHC [33,34,35,36,37,38], phosphorus-based neculeophiles [39,40] and phosphazene superbases [39,41,42,43,44] have been demonstrated be efficient catalysts for living GTP of acrylic monomers. On the other hand, Brønsted acids have been employed as activators for GTP as well [45], while GTP of acrylates catalyzed by Lewis acidic catalysts such as zinc halide [46], mercury iodide [47,48], or organoaluminun [46] requires a much higher catalyst loading (typically 10 mol % based on monomer) to achieve a reasonable degree of polymerization control. Through the oxidative activation of SKAs with a catalytic amount of [Ph_3_C][B(C_6_F_5_)_4_] (TTPB) as low as 0.025 mol % (based on monomer), we have successfully achieved high-speed living GTP of polar vinyl monomers catalyzed by the silylium ion R_3_Si^+^, including (meth)acrylates [49,50] and renewable MBL and MMBL [51]. Covalently linked, unimolecular silyl enolate/silylium nucleophile/electrophile bifunctional active species rendered a rate enhancement by a factor of >40 and high stereoselectivity (at low temperature), as compared to the mononuclear SKA system [52]. More recently, a tandem (FLP and LA) activation method was developed for GTP involving the in-situ generation of SKA initiators by 1,4-hydrosilylation of a methacrylate monomer. In such a process, the catalysts, such as highly electron deficient silylium cations “R_3_Si^+^” [53] or strong LAs E(C_6_F_5_)_3_ (E = B, Al) [5,6,54,55], play dual role in both hydrosilylation (via frustrated Lewis Pair (FLP)-type activation) and activation of monomer (classical LA activation). However, such system was shown to be much less effective for E(C_6_F_5_)_3_ (E = B, Al)-catalyzed GTP at high monomer to initiator ratios [6,53] and ill-controlled for Al(C_6_F_5_)_3_-catalyzed MMA polymerization. In this context, herein we report the SKA/B(C_6_F_5_)_3_ FLP-catalyzed GTP of polar vinyl monomers at room temperature, including MMA, renewable MMBL and MBL. More specifically, a highly interacting *^i^*^Bu^SKA/B(C_6_F_5_)_3_ LP system promotes the living GTP of MMBL and copolymerization of MMBL and MBL to produce well-defined (co)polymers with predicted molecular weights, and narrow molecular weight distributions. More importantly, this SKA/B(C_6_F_5_)_3_ LP system enabled us to isolate key reaction intermediates and perform kinetic and mechanistic studies, thereby providing the much-needed insights into the polymerization mechanism.

## 2. Results and Discussion

### 2.1. Slow MMA Polymerization by R_3_SiH/B(C_6_F_5_)_3_ and SKA/B(C_6_F_5_)_3_

**Scheme 1:**
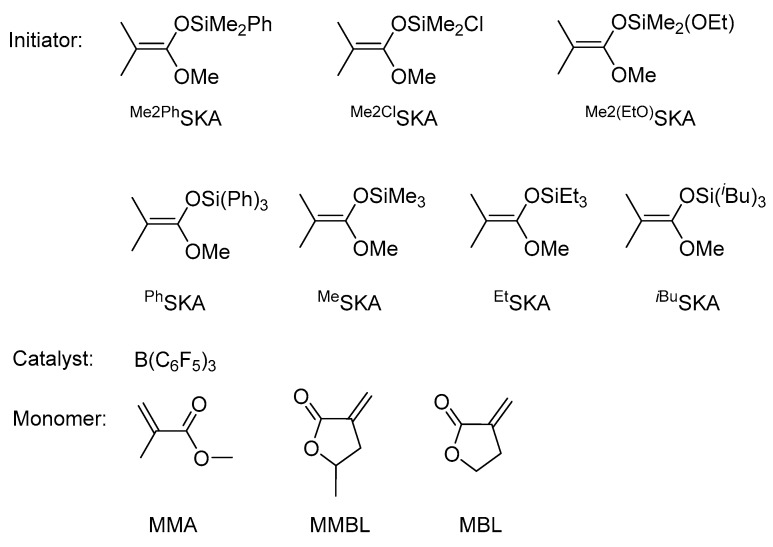
Chemical structures of initiators, catalyst and monomers employed in this study.

Without B(C_6_F_5_)_3_, all six hydrosilanes, including Et_3_SiH, Ph_3_SiH, *^i^*Bu_3_SiH, Me_2_ClSiH, Me_2_EtSiH, and Me_2_PhSiH, showed negligible activity for the polymerization of MMA in CH_2_Cl_2_ at room temperature. In the presence of B(C_6_F_5_)_3_, the in-situ generation of SKA initiators from the B(C_6_F_5_)_3_-catalyzed hydrosilylation of the monomer MMA with R_3_SiH exhibited very low polymerization activity and incomplete monomer conversions for various ratios of [MMA]_0_/[R_3_SiH]_0_/[B(C_6_F_5_)_3_]_0_ for up to 24 h (runs 1–18, Table S1). On the other hand, the direct use of SKA (Scheme 1) as initiator and B(C_6_F_5_)_3_ as catalyst did not lead to obvious enhancement in MMA polymerization activity compared to R_3_SiH/B(C_6_F_5_)_3_ systems (runs 19–39, Table S1). The corresponding PMMA polymers all possessed syndio-biased tacticities with a methyl triad distribution of around 70% rr, 28% mr and 2% mm, due to the chain-end control nature of this polymerization system.

### 2.2. Controlled MMBL Polymerization by R_3_SiH/B(C_6_F_5_)_3_ and SKA/B(C_6_F_5_)_3_

Next, we investigated the effectiveness of the R_3_SiH/B(C_6_F_5_)_3_ and SKA/B(C_6_F_5_)_3_ systems for renewable monomer MMBL, which is a cyclic analogue of MMA and is readily prepared in a two-step process from the cellulosic biomass-derived levulinic acid. Compared to the MMA polymerization, all R_3_SiH/B(C_6_F_5_)_3_ and SKA/B(C_6_F_5_)_3_ systems are more effective and controlled for polymerization of MMBL, achieving noticeably better initiation efficiency and narrower molecular weight distribution (Table 1 vs. Table S1). Among the silanes screened, Me_2_EtSiH and Me_2_PhSiH achieved quantitative monomer conversion within 30 min (*M*_n_ = 48.1 kg·mol^−1^, *Ð* = 1.26, I* = 47% for run 1, Table 1; *M*_n_ = 57.4 kg·mol^-1^, *Ð* = 1.39, I* = 39% for run 2, Table 1). Replacing the Ph group in the R_3_SiHstructure with the electron-withdrawing Cl (i.e., Me_2_ClSiH) rendered the polymerization reaching completion in 2 h and producing PMMBL with a *M*_n_ of 78.6 kg·mol^−1^ and a higher *Ð* value of 1.44 (run 3, Table 1). Interestingly, R_3_SiH with three Et substituents, Et_3_SiH, when combined with B(C_6_F_5_)_3_, exhibited comparable polymerization activity with that for Me_2_EtSiH, producing PMMBL with a *M*_n_ of 33 kg·mol^−1^ and a *Ð* value of 1.27, thus yielding an enhanced initiation efficiency of 68% (run 4, Table 2). With the increase of steric hindrance of substituents, the corresponding R_3_SiH exhibited drastically decreased polymerization activity (6 h for Ph_3_SiH and 24 h for *^i^*Bu_3_SiH to reach full monomer conversion) and initiation efficiency (21% for Ph_3_SiH and 7% for *^i^*Bu_3_SiH).

When switching to the SKA/B(C_6_F_5_)_3_ system, both ^Me2Ph^SKA/B(C_6_F_5_)_3_ and ^Me2(E^^tO)^SKA/B(C_6_F_5_)_3_ system achieved quantitative monomer conversion in 30 min and similar initiation efficiency (50% vs. 54%) under our current standard polymerization conditions {[MMBL]_0_:[SKA]_0_:[B(C_6_F_5_)_3_]_0_ = 200:1:1, 0.25 mL MMBL, 2.25 mL CH_2_Cl_2_, RT}. Replacing the phenyl group with a chlorine atom, the rate of MMBL polymerization was drastically decreased and quantitative monomer conversion was obtained in 6 h for ^Me2Cl^SKA/B(C_6_F_5_)_3_ system, producing PMMBL with a *M*_n_ of 85.1 kg·mol^−1^, a broader *Ð* value of 1.82 (Table 1, run 9), and thus giving a lower I*% of 27. The combination of ^Ph^SKA with B(C_6_F_5_)_3_ produced a polymerization system that is comparable with that of ^Me2Cl^SKA/B(C_6_F_5_)_3_ (run 10 vs. 9, Table 1). Interestingly, by replacing the three Ph groups with three electron-donating alkyl groups in SKA, we observed a significantly enhanced initiation efficiency I* (%) (71 for ^Me^SKA, run 11; 61 for ^Et^SKA, run 12; 70 for *^i^*^Bu^SKA, run 13).

Since both ^Me^SKA/B(C_6_F_5_)_3_ and *^i^*^Bu^SKA/B(C_6_F_5_)_3_ system exhibited the highest initiation efficiency for the polymerization of MMBL (around 70%) under our current condition, therefore, we employed both systems to examine their efficacies for polymerization of the homologues of MMBL, MBL or tulipalin A, which is a natural substance found in tulips and the MBL ring is an integral building block of many natural products. For polymerization with a 200:1:1 [MBL]:[^Me^SKA]:[B(C_6_F_5_)_3_] ratio, it took 24 h for both ^Me^SKA/B(C_6_F_5_)_3_ and *^i^*^Bu^SKA/B(C_6_F_5_)_3_ system to reach complete monomer consumption. However, it should be noted that PMBL produced by ^Me^SKA/B(C_6_F_5_)_3_ system exhibited a bimodal MWD. For polymerization with 100:1:1 [MBL]:[SKA]:[B(C_6_F_5_)_3_] ratio, only 30 min is needed to achieve quantitative monomer conversion for both systems, affording PMBL with predicted *M*_n_ and small *Ð* values (^Me^SKA: *M*_n_ = 12.7 kg·mol^−1^, *Ð* = 1.12, I* = 78%, Run 16; *^i^*^Bu^SKA: *M*_n_ = 13.4 kg·mol^−1^, *Ð* = 1.05, I* = 74%, Run 18, Table 1). These results indicated *^i^*^Bu^SKA/B(C_6_F_5_)_3_ system exhibited better control on the polymerization than that for ^Me^SKA/B(C_6_F_5_)_3_ system.

**Figure 1:**
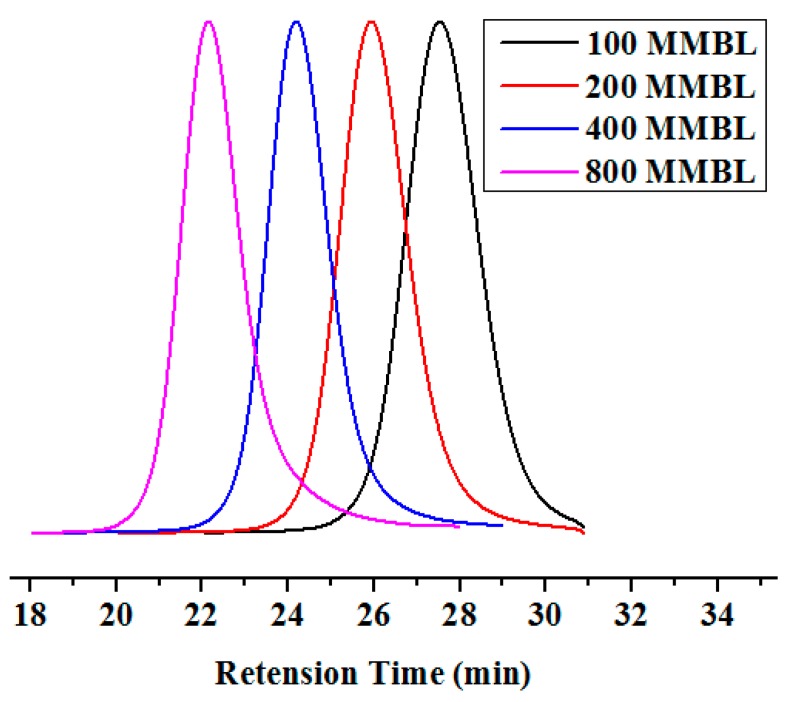
GPC traces of PMMBL samples produced by *^i^*^Bu^SKA/B(C_6_F_5_)_3_ with various [MMBL]_0_/[*^i^*^Bu^SKA]_0_ ratios at RT. Conditions: [MMBL]_0_/[*^i^*^Bu^SKA]_0_/[B(C_6_F_5_)_3_]_0_ = 400:4:1, 400:2:1, 400:1:1, 400:0.5:1, [MMBL]_0_ = 0.936 M.

**Figure 2:**
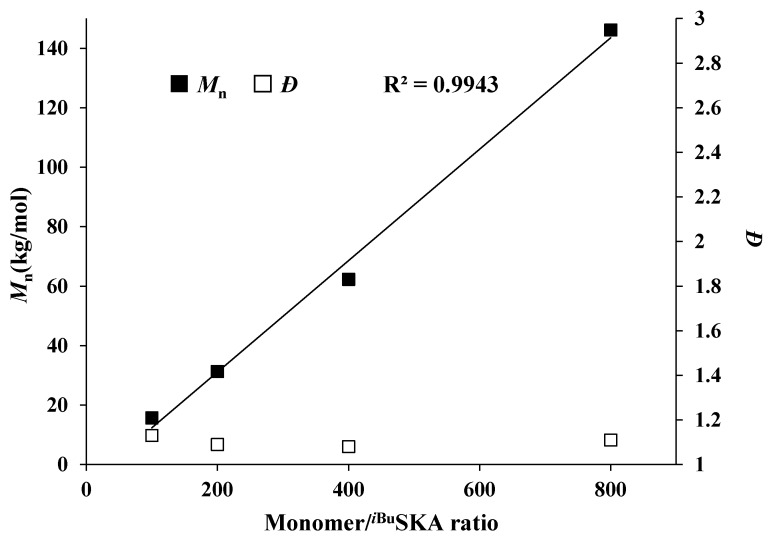
Plots of *M*_n_ and *Ð* values of PMMBL samples vs [MMBL]_0_/[*^i^*^Bu^SKA]_0_ ratio at RT. Conditions: [MMBL]_0_/[*^i^*^Bu^SKA]_0_/[B(C_6_F_5_)_3_]_0_ = 400:4:1, 400:2:1, 400:1:1, 400:0.5:1, [MMBL]_0_ = 0.936 M.

**Figure 3:**
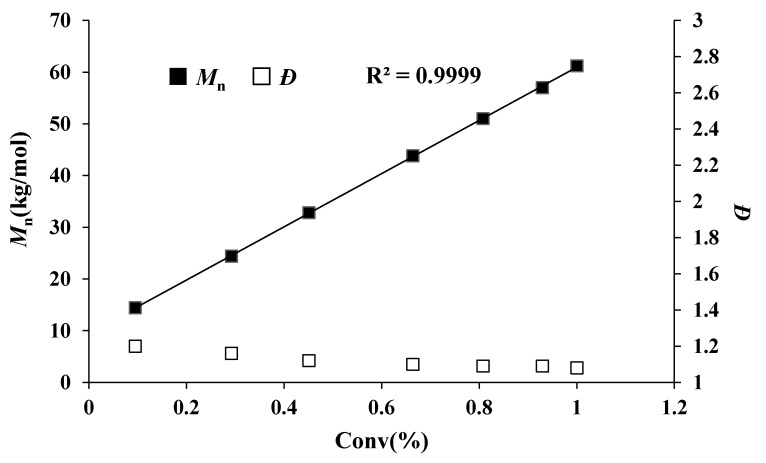
Plots of *M*_n_ and *Ð* values of PMMBL samples vs MMBL conversion by *^i^*^Bu^SKA/B(C_6_F_5_)_3_ system with MMBL/*^i^*^Bu^SKA/B(C_6_F_5_)_3_ = 400/1/1 ratio at RT.

In fact, the MMBL polymerization by *^i^*^Bu^SKA/B(C_6_F_5_)_3_ system is living and controlled, and near quantitative monomer conversion was achieved for polymerization with varied [MMBL]/[*^i^*^Bu^SKA] ratio from 100 to 800. GPC traces of PMMBL produced by *^i^*^Bu^SKA/B(C_6_F_5_)_3_ system also exhibited the gradual shift to the high-molar-mass region with an increase in the [M]/[I] ratio from 100 to 800 and maintained a narrow and unimodal MWD (Figure 1). Although increasing the catalyst loading of B(C_6_F_5_)_3_ would enhance the polymerization rate, the polymer MW is dependent on the concentration of initiator [*^i^*^Bu^SKA] and independent on the concentration of catalyst B(C_6_F_5_)_3_ (*vide infra*), which is evidenced by the linear increase of the *M*_n_ values of PMMBL produced by *^i^*^Bu^SKA/B(C_6_F_5_)_3_ system with an increase in the [MMBL]/[*^i^*^Bu^SKA] ratio from 100 to 800 (Figure 2, *R*^2^ = 0.994), while *Ð* values remained in the very narrow range of 1.08 to 1.13 (Figure 2). It is noted that *M*_n_ value of PMMBL obtained for polymerization with different [MMBL]/[B(C_6_F_5_)_3_] ratio maintained around 60 kg·mol^−1^ (Figure S21), which revealed that the concentration of [B(C_6_F_5_)_3_] has an effect the polymerization rate, but not on both polymer MW and MWD. Therefore, B(C_6_F_5_)_3_ is not the initiator but the catalyst for the activation of monomers. In addition to the aforementioned linearly increased polymer MW with the increase of varied monomer to initiator ratio, the living characteristics of MMBL polymerization by *^i^*^Bu^SKA/B(C_6_F_5_)_3_ was also confirmed by a plot of the PMMBL *M*_n_ vs monomer conversion at a fixed [MMBL]/[*^i^*^Bu^SKA]/[B(C_6_F_5_)_3_] ratio of 400:1:1, which clearly show a straight line (*R*^2^ = 0.999) with very narrow *Ð* value in the range of 1.08–1.2 (Figure 3).

### 2.3. Characterization of Key Intermediates

**Scheme 2:**
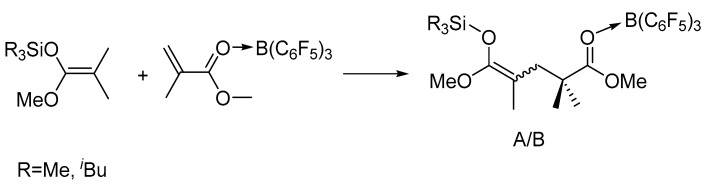
Generation of first-monomer-addition intermediate or active propagating species (**A** or **B** denotes **Z**/**E** isomers).

**Figure 4:**
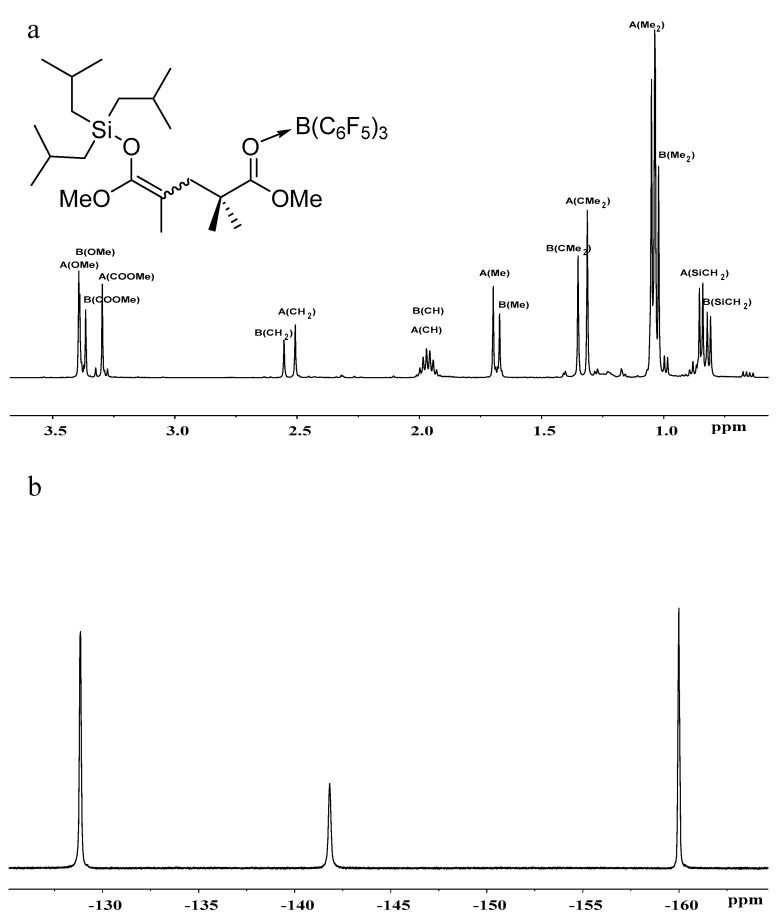
(**a**) ^1^H- and (**b**) ^19^F-NMR spectra of *^i^*Bu_3_SiO(OMe)C=C(Me)CH_2_CMe_2_C(OMe)=O···B(C_6_F_5_)_3_.

To gain more insights into the polymerization, we studied the in situ NMR reactions of ^Me^SKA with B(C_6_F_5_)_3_ in 1:1 ratio and observed the formation of FLP, which is evidenced by the fact that there is no obvious interaction observed in the reaction of ^Me^SKA with B(C_6_F_5_)_3_ (Figures S11 and S12). Similar FLP was generated in the reaction of *^i^*^Bu^SKA with B(C_6_F_5_)_3_ in 1:1 ratio (Figures S13 and S14), too. We also prepared B(C_6_F_5_)_3_·MMA (Figures S7 and S8) and B(C_6_F_5_)_3_·MMBL (Figures S9 and S10) adduct for the study of their reaction with SKA, respectively. The reaction of ^Me^SKA with B(C_6_F_5_)_3_·MMA at room temperature in C_6_D_6_ generated major products of dimeric SKA analogue Me_3_SiO(OMe)C=C(Me)CH_2_CMe_2_C(OMe)=O· · ·B(C_6_F_5_)_3_ as two isomers (Z/E) in a 3:2 ratio due to the cis-trans isomerism of MMA (Scheme 2), which could readily derived from the analysis of ^1^H- and ^13^C-NMR spectra (Figures S15 and S17).The characteristic proton signals at 0.18 ppm for major isomer and 0.16 ppm for minor isomer attributed to the trimethyl substituents of silyl group in the dimeric SKA analogue is the result of the high field shift from the original 0.18 ppm for neutral ^Me^SKA, which is different from that for Me_3_Si^+^ generated by vinylogous hydride abstraction of ^Me^SKA with Ph_3_C^+^, exhibiting signal at 0.65 ppm [49]. This result suggests that the silyl group in the dimeric SKA analogue was neutral rather than Me_3_Si^+^. In combination with the fact that ^19^F NMR spectrum exhibiting similar signals [δ −129.03 (br, 6F, *o*-F), −142.24 (br, 3F, *p*-F), −160.09 (s, 6F, *m*-F))] (Figure S16) with that for B(C_6_F_5_)_3_·MMA adduct (Figure S8), we proposed the structure for corresponding dimeric SKA analogues as shown in Scheme 2. Similarly, the reaction of *^i^*^Bu^SKA with B(C_6_F_5_)_3_·MMA cleanly afforded the single-monomer-addition product (Scheme 2), as revealed by the ^1^H-, ^1^^9^F- and ^13^C-NMR spectra (Figure 4a,b, and Figure S20). However, we did not observe the formation of such dimeric SKA analogue for reaction of SKA with B(C_6_F_5_)_3_·MMBL probably due to high reactivity of MMBL.

### 2.4. Kinetics and Mechanism of Polymerization

**Figure 5:**
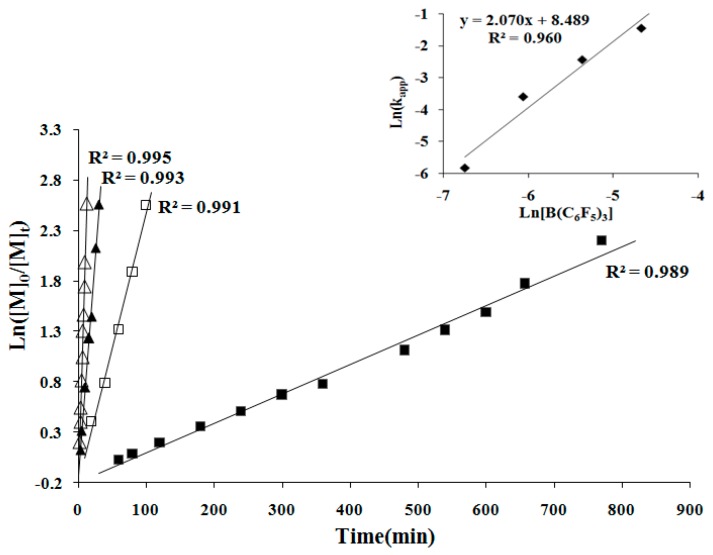
First-order kinetic plots for MMBL polymerization by *^i^*^Bu^SKA/B(C_6_F_5_)_3_ in CH_2_Cl_2_ at RT: [MMBL]_0_ = 0.936 M; [SKA]_0_ = 2.34 mM; [B(C_6_F_5_)_3_]_0_ = 9.36 mM (∆), 4.68 mM (▲), 2.34 mM (□), 1.17 mM (■). Inset: plot of ln(*k*_app_) vs. ln[B(C_6_F_5_)_3_].

To continue the investigation of the mechanistic aspects of the polymerization, we next examined the kinetics of the MMBL polymerization by the [*^i^*^Bu^SKA]/[B(C_6_F_5_)_3_]. The kinetic experiments employed [MMBL]_0_/[*^i^*^Bu^SKA]_0_/[B(C_6_F_5_)_3_]_0_ with varied ratio of 400:1:0.5, 400:1:1, 400:1:2, 400:1:4 and a fixed [MMBL]_0_/[*^i^*^Bu^SKA]_0_ ratio of 400. As can be seen from the representative kinetic plots of Ln([MMBL]_0_/[MMBL]*_t_*) vs. time, the polymerization clearly showed a first-order dependence on [MMBL] for all ratios investigated (Figure 5). Furthermore, a double logarithm plot (insert) of the apparent rate constants (*K*_app_) obtained from the slopes of the best-fit lines to the plots of Ln([MMBL]_0_/[MMBL]*_t_*) vs. time as a function of ln[B(C_6_F_5_)_3_] was fit to a straight line (*R*^2^ = 0.960) with a slop of 2.07. Therefore, the kinetic order with respect to the [B(C_6_F_5_)_3_], given by the slopes of ~2, reveals that the polymerization is second-order in B(C_6_F_5_)_3_ catalyst concentration; these kinetics results suggest a bimolecular propagation.

**Figure 6:**
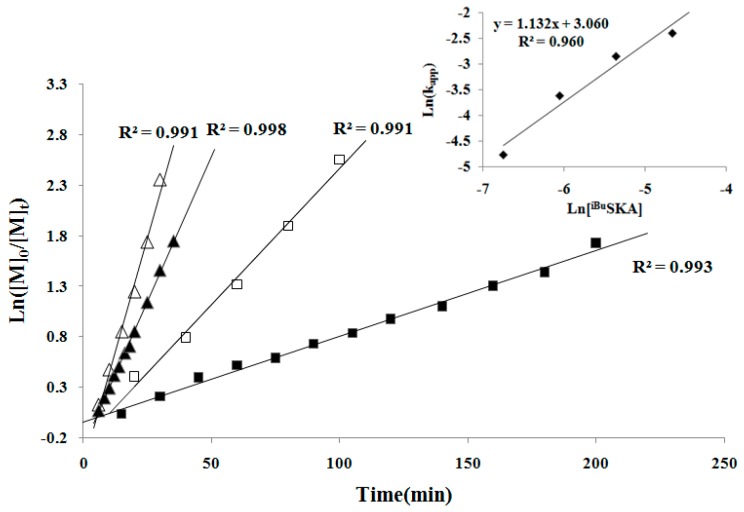
First-order kinetic plots for MMBL polymerization by *^i^*^Bu^SKA/B(C_6_F_5_)_3_ in CH_2_Cl_2_ at RT: [MMBL]_0_ = 0.936 M; [B(C_6_F_5_)_3_]_0_ = 2.34 mM; [SKA]_0_ = 9.36 mM (∆), 4.68 mM (▲), 2.34 mM (□), 1.17 mM (■). Inset: plot of ln(*k*_app_) vs. ln[*^i^*^Bu^SKA].

In the second set of kinetic experiments, with a fixed [MMBL]_0_/[B(C_6_F_5_)_3_]_0_ ratio of 400, the kinetic experiments was carried out with varied [MMBL]_0_/[B(C_6_F_5_)_3_]_0_/[*^i^*^Bu^SKA]_0_ ratio of 400:1:0.5, 400:1:1, 400:1:2, 400:1:4. The same first-order dependence was observed for all ratios investigated in this study (Figure 6). A double logarithm plot (insert) of the apparent rate constants (*k*_app_), obtained from the slopes of the best-fit lines to the plots of Ln([MMBL]_0_/[MMBL]*_t_*) vs. time, as function of ln[*^i^*^Bu^SKA] was fit to a straight line (*R*^2^ = 0.960) with a slop of 1.132, revealing that the propagation is first-order in the concentration of [*^i^*^Bu^SKA]. Overall, the polymerization by the [*^i^*^Bu^SKA]/[B(C_6_F_5_)_3_] systems follows a bimolecular, activated monomer propagation mechanism.

**Scheme 3:**
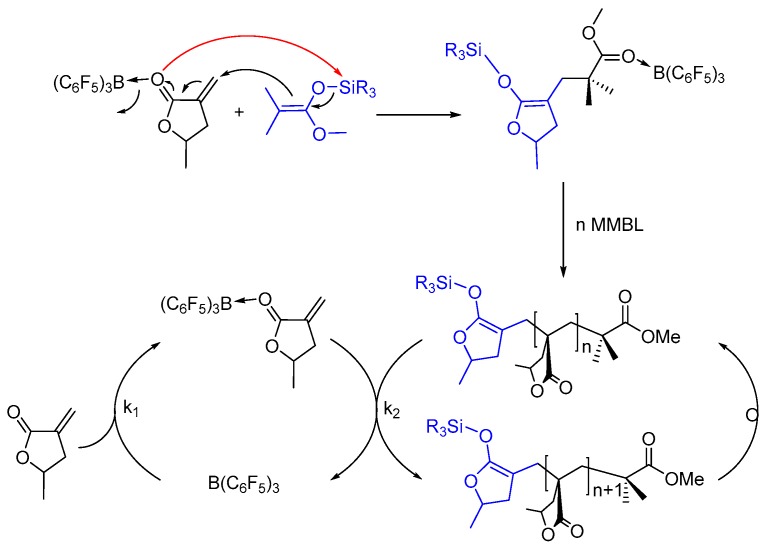
Proposed mechanism for MMBL polymerization by SKA/B(C_6_F_5_)_3_.

On the basis of the above kinetic results, coupled with mechanistic insights obtained through monitoring the polymerization and characterization of the reaction intermediates (*vide supra*), we propose the following initiation and propagation mechanism for polymerization of polar vinyl monomers by the SKA/B(C_6_F_5_)_3_ LP system taking MMBL as example (Scheme 3). In this mechanism, the reaction was initiated with the intermolecular Michael addition of the ester enolate groups of the SKA to the vinyl group of B(C_6_F_5_)_3_-activated monomer, meanwhile the Si-O bond of SKA was cleaved and the silyl group was transferred to the carbonyl group of the B(C_6_F_5_)_3_-activated monomer to generate the single-monomer-addition species or the active propagating species. In the propagation cycle, the B(C_6_F_5_)_3_ catalyst is released from the propagating chain to the incoming monomer, followed by the subsequent intermolecular Michael addition to generate the polymeric SKA intermediate (Scheme 3). Our kinetic studies indicated that the polymerization is first-order dependent on both monomer and *^i^*^Bu^SKA initiator concentration, but second-order dependent on B(C_6_F_5_)_3_ catalyst concentrations, which revealed that the rate (*k*_2_) of the intermolecular Michael addition of the SKA or its homologues to the B(C_6_F_5_)_3_-activated monomer is comparable with that (*k*_1_) for the release of the catalyst B(C_6_F_5_)_3_ from the ester group of the growing polymer chain to the incoming monomer for monomer activation (*k*_1_ ≈ *k*_2_).

**Figure 7:**
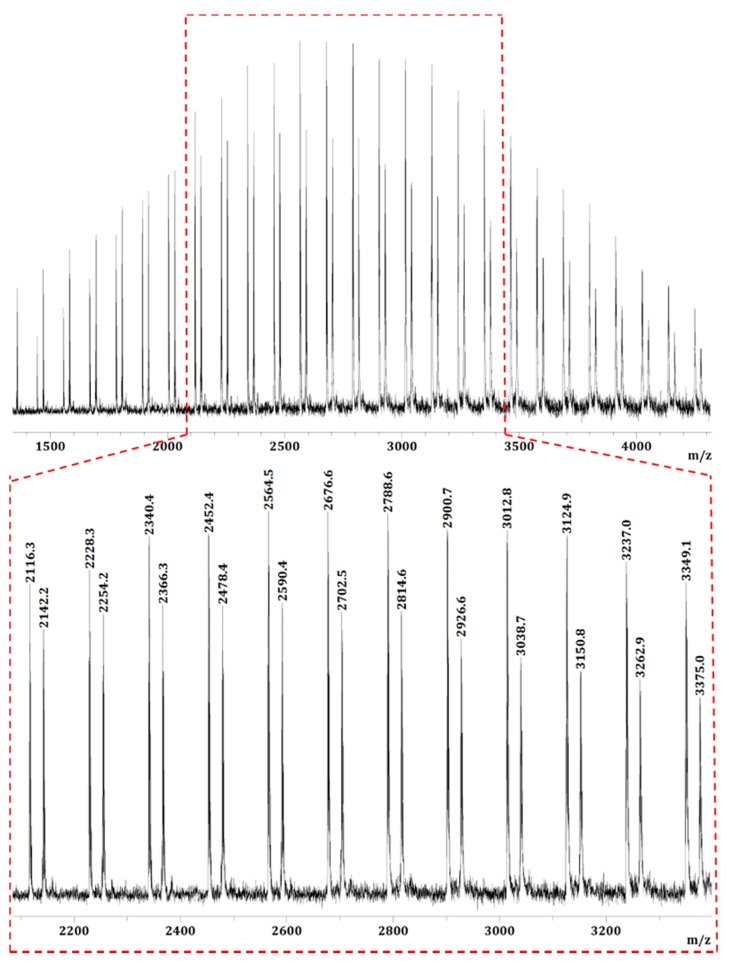
MALDI-TOF mass spectrum of the low-MW PMMBL sample produced by *^i^*^Bu^SKA/B(C_6_F_5_)_3_ in CH_2_Cl_2_ at RT.

**Figure 8:**
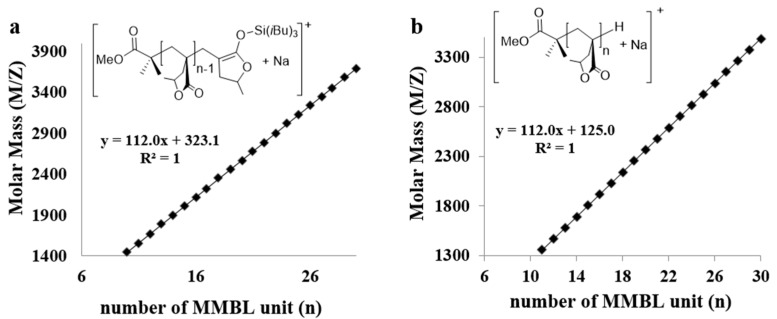
Plot of *m*/*z* values from Figure 7 vs. the number of MMBL repeat units (*n*) for major series (**a**) and minor series (**b**).

The low MW PMMBL produced by the *^i^*^Bu^SKA/B(C_6_F_5_)_3_ system was analyzed by matrix-assisted laser desorption/ionization time of flight mass spectroscopy (MALDI-TOF MS). As can be seen from Figure 7, The MS spectrum consisted of two series of molecular mass ions. A plot (Figure 8a) of *m/z* values for the major series vs the number of MMBL repeat units (*n*) yielded a straight line with a slope of 112 (mass of MMBL) and an intercept of 323 corresponding to the sum of end groups: MeOC(=O)C(Me)_2_/Si(*i*Bu)_3_ + Na^+^. This analysis yielded a polymer chain structure of MeOC(=O)C(Me)_2_-(MMBL)*_n_*-Si(*i*Bu)_3_, with both the initiating (the ester enolate group) and termination (the silyl group) chain ends being derived from *^i^*BuSKA. Furthermore, a linear plot of *m*/*z* values for the minor series in the MS spectrum vs the MMBL repeat units (*n*) gave the same slope but a different intercept of 125 (Figure 8b), which corresponds to the sum of end groups: MeOC(=O)C(Me)_2_-(MMBL)*_n_*-H + Na^+^. Formation of such enolate/H chain end is presumably a result of desilylation during the preparation of the MALDI-TOF sample.

### 2.5. Random and Block Copolymerizations of MMBL with MBL

**Figure 9:**
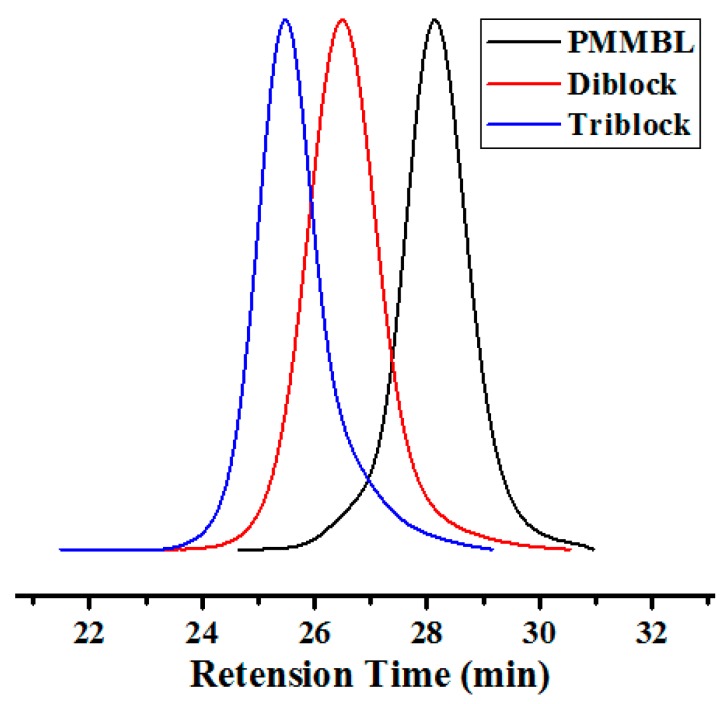
GPC traces of homopolymer PMMBL (black), diblock copolymer PMMBL-*b*-PMBL (red), and triblock copolymer PMMBL-*b*-PMBL-*b*-PMMBL (blue) produced by *^i^*^Bu^SKA/B(C_6_F_5_)_3_ in CH_2_Cl_2_ at RT.

The living features of polymerization of MMBL and MBL by *^i^*^Bu^SKA/B(C_6_F_5_)_3_ system, as demonstrated by the above described experiments, also enabled the synthesis of well-defined copolymers. As shown in Table 2, when both monomers were added simultaneously, the copolymerization of MBL and MMBL produced a randomly sequenced copolymer with *M*_n_ = 28.3 kg·mol^−1^ and *Ð* = 1.03 (run 1, Table 2, Figure S23). On the other hand, sequential block copolymerization by polymerizing MMBL first with [MMBL]/[*^i^*^Bu^SKA]/[B(C_6_F_5_)_3_]=100:1:1 without quenching, followed by addition of another 100 equiv. of MBL, successfully afforded linear diblock copolymer PMMBL-*b*-PMBL. As can be seen from Figure 9, the GPC traces for the PMMBL produced during the initial MMBL polymerization shifted to a higher molecular weight region with low MWD value of 1.07, while the *M*_n_ increase from 13.2 kg·mol^−1^ (black trace) for the homopolymer PMMBL to 25.7 kg·mol^−1^ (red trace) for the diblock copolymer PMMBL-*b*-PMBL (run 2, Table 2), which provided further evidence for the formation of well-defined block copolymer by *^i^*^Bu^SKA/B(C_6_F_5_)_3_ system. Through the same sequential monomer addition method, well-defined triblock copolymer PMMBL-*b*-PMBL-*b*-PMMBL was also successfully prepared with a *M*_n_ value of 37.1 kg/mol and a *Đ* value of 1.06 (Figure 9, blue trace, run 3, Table 2). We also characterized both block copolymers and random copolymers by ^13^C-NMR analysis (Figure S21). In contrast to diblock copolymer PMMBL-*b*-PMBL showing two signals corresponding to the PMBL and PMMBL, the yielded random copolymer PMMBL-*r*-PMBL only exhibited one broad peak in the carbonyl signals, which is ranging between the signals for homopolymers PMBL and PMMBL. The combination of such conclusive ^13^C NMR results and GPC analyses should provide sufficient evidence for the formation of random and block copolymer from statistical and sequential block polymerization, respectively.

## 3. Metarials and Methods

### 3.1. Materials, Reagents and Methods

All syntheses and manipulations of air- and moisture-sensitive materials were carried out in flamed Schlenk-type glassware on a dual-manifold Schlenk line, or an inert gas-filled glovebox. Toluene, benzene, THF and hexane were refluxed over sodium/potassium alloy distilled under nitrogen atmosphere, then stored over molecular sieves 4 Å. CH_2_Cl_2_ was refluxed over CaH_2_ distilled under nitrogen atmosphere, then stored over molecular sieves 4 Å. Benzene-*d*_6_ was dried over molecular sieves 4 Å. NMR spectra were recorded using an Avance II 500 (500 MHz, ^1^H; 126 MHz, ^13^C; 471 MHz, ^19^F) instrument (Bruker, Karlsruhe, Baden-Württemberg, Germany) in appropriate deuterated solvents at room temperature. Chemical shifts for ^1^H and ^13^C spectra were referenced to internal solvent resonances and are reported as parts per million relative to SiMe_4_, whereas ^19^F-NMR spectra were referenced to external CFCl_3_. Air sensitive NMR samples were conducted in Teflon-valve sealed J. Young-type NMR tubes.

Methyl methacrylate (MMA) was purchased from J&K (Beijing, China), while α-methylene-γ-butyrolactone (MBL) and γ-methyl-α-methylene-γ-butyrolactone (MMBL) were purchased from TCI (Tokyo, Japan). These monomers were first degassed by three freeze-pump-thaw cycles and dried over CaH_2_ for 12 h, followed by vacuum distillation. Further purification of MMA involved titration with tri(*n*-octyl)aluminum (Strem Chemicals, Newburyport, MA, USA) to a yellow end point [56], followed by distillation under reduced pressure. All purified monomers were stored in brown bottles inside a glovebox freezer at −30 °C. Dimethylphenylsilane (Me_2_PhSiH), and triphenylsilane (Ph_3_SiH) were purchased from TCI. Triisobutylsilane (*i*Bu_3_SiH), dimethylethylsilane (Me_2_EtSiH), dimethylketene methyl trimethylsilyl acetal (^Me^SKA) were purchased from Sigma-Aldrich (St. Louis, MO, USA). Butylated hydroxytoluene (BHT-H, 2,6-di-*tert*-butyl-4-methylphenol), triethylsilane (Et_3_SiH), chlorodimethyl-silane (Me_2_ClSiH), bromopentafluorobenzene were purchased from J&K. Boron trichloride (1.0 M solution in hexanes) and *n*-BuLi (2.5 M solution in hexanes) were purchased from Energy Chemical (Shanghai, China). Tris(pentafluorophenyl)borane, B(C_6_F_5_)_3_, was prepared according to literature procedures.[57,58] Literature procedures were also employed for the preparation of the following compounds: dimethylketene methyl triethylsilyl acetal (^Et^SKA) [49], dimethylketene methyl triphenylsilyl acetal Me_2_C=C(OMe)OSiPh_3_ (^Ph^SKA) [50], dimethylketene nethyl triisobutylsilyl acetal Me_2_C=C(OMe)OSi(^i^Bu)_3_ (*^i^*^Bu^SKA) [50],dimethylketene methyl dimethyl-phenylsilyl acetal Me_2_C=C(OMe)OSiMe_3_ (^Me2Ph^SKA) [54].

### 3.2. Synthesis of Dimethylketene Methyl Chlorodimethylsilyl Acetal Me_2_C=C(OMe)OSiMe_2_Cl (^Me2Cl^SKA)

In an inert gas-filled glovebox, solution containing B(C_6_F_5_)_3_ (128 mg, 0.250 mmol) and MMA (2.65 mL, 25.0 mmol) in dry CH_2_Cl_2_ (50 mL) was charged in a 100 mL Schlenk flask equipped with a stir bar. This flask was sealed with a rubber septum, removed from the glovebox, interfaced to a Schlenk line, and brought to −78 °C for at least 15 min. Chlorodimethylsilane (2.78 mL, 25.0 mmol) was added dropwise to the above flask under nitrogen atmosphere. The resulting reaction mixture was allowed to warm up to room temperature slowly and then subjected to vacuum. The residue was purified by vacuum distillation to afford the final product as colorless oil. Yield: 3.84 g (79%).^1^H-NMR (500 MHz, benzene-*d*_6_) δ 3.35 (s, 3H, O*Me*), 1.64 (s, 3H, =C*Me*), 1.61 (s, 3H, =C*Me*), 0.36 (s, 6H, Si*Me*_2_Cl). ^13^C-NMR (126 MHz, benzene-*d*_6_) δ 149.2, 92.8, 57.2, 17.1, 16.4, 2.3.

### 3.3. Synthesis of Chloro(ethoxy)dimethylsilane Me_2_(EtO)SiCl

Literature procedures [59] were modified for the preparation of Me_2_(EtO)SiCl. In an inert gas-filled glovebox, solution of B(C_6_F_5_)_3_ (461 mg, 0.90 mmol) and EtOH (5.08 mL, 90.0 mmol) in dry CH_2_Cl_2_ (100 mL) was charged in a 200 mL Schlenk flask equipped with a stir bar. This flask was sealed with a rubber septum, removed from the glovebox, interfaced to a Schlenk line, and brought to −78 °C for at least 20 min. Chlorodimethylsilane(10.0 mL, 90.0 mmol) was added dropwise to the flask under nitrogen atmosphere. The resulting mixture was allowed to warm up to room temperature slowly and then subjected to vacuum. The residue was purified by distillation to afford the product as colorless oil. Yield: 12.48 g (91%).^1^H-NMR (500 MHz, benzene-*d*_6_) δ 3.61 (q, *J* = 7.0 Hz, 2H, OC*H*_2_), 1.06 (t, *J* = 7.0 Hz, 3H, OCH_2_C*H*_3_), 0.26 (s, 6H, Si*Me*_2_). ^13^C-NMR (126 MHz, benzene-*d*_6_) δ 59.1, 18.1, 2.0.

### 3.4. Synthesis of Dimethylketene Methyl Ethoxydimethylsilyl Acetal Me_2_C=C-(OMe)OSiMe_2_(EtO) (^Me2(EtO)^SKA)

Literature procedures for the general synthesis of ketene trialkylsilyl acetals [60,61] were modified for the preparation of ^Me2(EtO)^SKA. In an inert gas-filled glovebox, a solution of diisopropylamine (7.05 mL, 5.06 g, 50.0 mmol) in dry THF (100 mL) was charged in a 200 mL Schlenk flask equipped with a stir bar. This flask was sealed with a rubber septum, removed from the glovebox, interfaced to a Schlenk line, and placed in a 0 °C ice-water bath for at least 15 min. *n*-Butyllithium (32.0 mL, 1.6 M in hexane, 51.2 mmol) was added dropwise to the above flask under nitrogen atmosphere. After stirring at 0 °C for 30 min, methyl isobutyrate (5.74 mL, 5.11 g, 50.0 mmol) was added slowly. The reaction mixture was stirred at this temperature for 30 min, after which Chloro(ethoxy)dimethylsilane (7.61 g, 50.0 mmol) was added. The mixture was allowed to warm slowly to room temperature and stirred overnight, after which all volatiles were removed in vacuum and hexanes (50 mL) was added. The resulting precipitates were filtered off under nitrogen atmosphere, the solvent of the filtrate was removed under reduced pressure. The residue was purified by distillation under vacuum to afford the product as colorless oil. Yield: 8.79 g (86%). ^1^H-NMR (500 MHz, benzene-*d*_6_) δ 3.72 (q, *J* = 7.0 Hz, 2H, OC*H*_2_CH_3_), 3.39 (s, 3H, O*Me*), 1.72 (s, 3H, =C*Me*), 1.68 (s, 3H, =C*Me*), 1.13 (t, *J* = 7.0 Hz, 3H, OCH_2_C*H*_3_), 0.21 (s, 6H, Si*Me*_2_). ^13^C-NMR (126 MHz, benzene-*d*_6_) δ 149.8, 90.7, 58.6, 56.6, 18.5, 17.1, 16.5, −2.5.

### 3.5. Isolation of Adduct B(C_6_F_5_)_3_·MMA

To a solution of B(C_6_F_5_)_3_ (512 mg, 1.0 mmol) in hexane (15 mL) was added MMA (110 mg, 1.1 mmol) at room temperature with reacting for 30 min. The solution was brought to −30 °C in glovebox freezer for 1 h. After filtration and removal of organic solvents in vacuo, the B(C_6_F_5_)_3_·MMA was obtained as a white powder (546 mg, 89%).^1^H-NMR (benzene-*d*_6_) δ 5.91 (s, 1H, =C*H*), 5.05–5.03 (s, 1H, =C*H*), 3.29 (s, 3H, O*Me*), 1.63 (s, 3H, *Me*).^19^F-NMR (benzene-*d*_6_) δ −130.18 (d, *J* = 20.2 Hz, 6F, *o*-F), −144.90 (t, *J* = 20.8 Hz, 3F, *p*-F), −160.70 (m, 6F, *m*-F).

### 3.6. Isolation of Adduct B(C_6_F_5_)_3_·MMBL

The B(C_6_F_5_)_3_·MMBL adduct was isolated as a white powder in 91% yield using the same procedure as described for the isolate of the adduct B(C_6_F_5_)_3_·MMA. ^1^H-NMR (500 MHz, benzene-*d*_6_) δ 6.06 (t, *J* = 3.0 Hz, 1H, =C*H*), 4.86 (t, *J* = 2.7 Hz, 1H, =C*H*), 3.67 (ddq, *J* = 8.0, 6.3, 5.7 Hz, 1H, OC*H*), 1.56 (ddt, *J* = 17.3, 8.0, 2.7 Hz, 1H, C*H*_2_), 1.10 (ddt, *J* = 17.3, 5.7, 2.9 Hz, 1H, C*H*_2_), 0.26 (d, *J* = 6.3 Hz, 3H, *Me*). ^19^F- NMR (471 MHz, benzene-*d*_6_) δ −134.81 (dd, *J* = 23.2, 7.3 Hz, 6F, *o*-F), −156.72 (t, *J* = 20.7 Hz, 3F, *p*-F), −163.93 (m, 6F, *m*-F).

### 3.7. NMR Reaction of Me_2_C=C(OMe)OSiMe_3_ (^Me^SKA) with B(C_6_F_5_)_3_

In an inert gas-filled glovebox, solution of ^Me^SKA (1.74 mg, 0.01 mmol) in 0.3 mL of C_6_D_6_ was charged in a Teflon-valve-sealed J. Young-type NMR tube. A 0.3 mL C_6_D_6_ solution of B(C_6_F_5_)_3_ (5.12 mg, 0.01 mmol) was added to this tube via pipet at room temperature and allowed to react for ~15 min before the NMR spectra were recorded, which showed there were no reaction between ^Me^SKA and B(C_6_F_5_)_3_. ^1^H-NMR (500 MHz, benzene-*d*_6_) δ 3.33 (s, 3H, O*Me*), 1.73 (s, 3H, =C*Me*), 0.65 (s, 3H, =C*Me*), 0.18 (s, 9H, Si*Me*_3_). ^19^F-NMR (471 MHz, benzene-*d*_6_) δ −128.82 (d, 21.7 Hz, 6F, *o*-F), −141.81 (t, *J* = 21.3 Hz, 3F, *p*-F), −160.06 (m, 6F, *m*-F).

### 3.8. NMR Reaction of ^Me^SKA with B(C_6_F_5_)_3_·MMA

In an inert gas-filled glovebox, solution of ^Me^SKA (1.74 mg, 0.01 mmol) in 0.3 mL of C_6_D_6_ was charged in a Teflon-valve-sealed J. Young-type NMR tube. A 0.3 mL C_6_D_6_ solution of B(C_6_F_5_)_3_·MMA (6.12 mg, 0.01 mmol) was slowly added to this tube via pipet at room temperature and allowed to react for ~15 min before the NMR spectra were recorded. The resulting mixture showed the clean formation of the species Me_3_SiO(OMe)C=C(Me)CH_2_CMe_2_C(OMe)=O···B(C_6_F_5_)_3_ (**1**) as two isomers (Z/E) in 3:2 ratio: major isomer **1A** and minor isomer **1B**, plus a small amount of unreacted starting materials. **1A**: ^1^H-NMR (500 MHz, benzene-*d*_6_) δ 3.395 (s, 3H, O*Me*), 3.25 (s, 3H, COO*Me*), 2.53(s, 2H, C*H*_2_), 1.65 (s, 3H, *Me*), 1.31 (s, 6H, *Me*_2_), 0.18 (s, 9H, Si*Me*_3_); **1B**: δ 3.392 (s, 3H, O*Me*), 3.30 (s, 3H, COO*Me*), 2.46 (s, 2H, C*H*_2_), 1.72 (s, 3H, *Me*), 1.32 (s, 6H, *Me*_2_), 0.16 (s, 9H, Si*Me*_3_); ^19^F-NMR (471 MHz, benzene-*d*_6_) δ −129.03 (br, 6F, *o*-F), −142.24 (br, 3F, *p*-F), −160.09 (s, 6F, *m*-F). ^13^C-NMR (126 MHz, benzene-*d*_6_) δ 178.30, 178.2, 153.3, 152.2, 149.4, 147.4, 138.7, 136.7, 92.1, 91.6, 57.2, 55.6, 51.4, 51.3, 43.14, 43.05, 42.5, 42.4, 25.8, 16.5, 15.4, 0.3, 0.1.

### 3.9. NMR Reaction of Me_2_C=C(OMe)OSi(^i^Bu)_3_ (^iBu^SKA) with B(C_6_F_5_)_3_

This reaction was carried out in the same manner as the reaction of ^Me^SKA with B(C_6_F_5_)_3_, which shown there were no reaction between *^i^*^Bu^SKA and B(C_6_F_5_)_3_. ^1^H-NMR (500 MHz, benzene-*d*_6_) δ 3.38 (s, 3H, O*Me*), 1.98 (sept, *J* = 6.6 Hz, 3H, C*H*Me_2_), 1.70 (s, 3H, =C*Me*), 1.69 (s, 3H, =C*Me*), 1.05 (d, *J* = 6.6 Hz, 18H, CH*Me*_2_), 0.84 (d, *J* = 6.8 Hz, 6H, SiC*H*_2_). ^19^F-NMR (471 MHz, benzene-*d*_6_) δ −128.82 (d, *J* = 21.7, 6F, *o*-F), −141.83 (t, *J* = 20.9 Hz, 3F, *p*-F), −160.07 (m, 6F, *m*-F).

### 3.10. NMR Reaction of ^iBu^SKA with B(C_6_F_5_)_3_·MMA

This reaction was carried out in the same manner as the reaction of ^Me^SKA with B(C_6_F_5_)_3_·MMA, forming cleanly *^i^*Bu_3_SiO(OMe)C=C(Me)CH_2_CMe_2_C(OMe)=O···B(C_6_F_5_)_3_ (**3**) as two isomers (Z/E) in 4:3 ratio: major isomer **2A** and minor isomer **2B**. **2A**: ^1^H-NMR (500 MHz, benzene-*d*_6_) δ 3.401 (s, 3H, O*Me*), 3.30 (s, 3H, COO*Me*), 2.52 (s, 2H, C*H*_2_), 1.98 (m, 3H, C*H*Me_2_), 1.71 (s, 3H, *Me*), 1.32 (s, 6H, C*Me*_2_), 1.05 (d, *J* = 6.5 Hz, 18H, CH*Me*_2_), 0.85 (d, *J* = 6.9 Hz, 6H, SiC*H*_2_); **2B**: δ 3.397 (s, 3H, O*Me*), 3.37 (s, 3H, COO*Me*), 2.56 (s, 2H, C*H*_2_), 1.98 (m, 3H, C*H*Me_2_), 1.68 (s, 3H, *Me*), 1.36 (s, 6H, C*Me*_2_), 1.04 (d, *J* = 6.8 Hz, 18H, CH*Me*_2_), 0.82 (d, *J* = 6.9 Hz, 6H, SiC*H*_2_); ^19^F-NMR (471 MHz, benzene-*d*_6_) δ −128.84 (br, 6F, *o*-F), −141.82 (br,3F,*p*-F), −159.99 (m, 6F, *m*-F). ^13^C-NMR (126 MHz, benzene-*d*_6_) δ 149.4, 147.4, 138.7, 136.7, 92.5, 58.6, 56.9, 51.3, 51.3, 43.0, 42.4, 42.3, 26.8, 26.7, 26.5, 26.4, 25.9, 25.7, 24.5, 16.6, 15.1.

### 3.11. General Polymerization Procedures

Polymerizations were performed in 20 mL glass reactors inside the inert gas-filled glovebox for ambient temperature (ca*.* 25 °C) runs. In a typical procedure, a predetermined amount of B(C_6_F_5_)_3_ and monomer (1 mL for MMA, 250 μL for MMBL or 205 μL for MBL, 200 equiv. relative to the SKA) were dissolved in dry CH_2_Cl_2_. A solution of a SKA (1 equiv. of a LA) in 1.0 mL of solvent were added rapidly via a gastight syringe to the solution of B(C_6_F_5_)_3_-monomer. The amount of the monomer was fixed for all polymerizations. After stirring for the measured time, a 0.1 mL aliquot was withdrawn from the reaction mixture and quickly quenched into 0.6 mL of undried “wet” CDCl_3_ stabilized by 250 ppm of BHT-H; the quenched aliquots were later analyzed by ^1^H-NMR to obtain the monomer conversion. After the polymerization was stirred for the stated reaction time then the reactor was taken out of the glovebox, and quenched by addition of 10 mL of 5% HCl-acidified methanol. The quenched mixture was isolated by filtration and dried in vacuo overnight at room temperature to a constant weight.

### 3.12. Polymerization Kinetics

Kinetic experiments were carried out in a stirred glass reactor at ambient temperature (ca*.* 25 °C) inside an inert gas-filled glovebox using the polymerization procedure already described above, with the [B(C_6_F_5_)_3_]/[*^i^*^Bu^SKA] ratio was 0.5/1, 1/1, 2/1, 4/1, [MMBL]_0_ was fixed at 0.936 M and [*^i^*^Bu^SKA]_0_ was fixed at 2.34 mM, where [B(C_6_F_5_)_3_] = 1.17, 2.34, 4.68, 9.36 mM in 2.5 mL mixture solutions. At appropriate time intervals, 0.1 mL aliquots were taken from the reaction mixture and quickly quenched into 0.6 mL of undried “wet” CDCl_3_ mixed with 250 ppm BHT-H. The quenched aliquots were analyzed by ^1^H-NMR for determining the [MMBL]*_t_* at a given time *t* and Ln([MMBL]_0_: [MMBL]*_t_*). Apparent rate constants (*k*_app_) were extracted from the slopes of the best fit lines to the plots of Ln([MMBL]_0_: [MMBL]*_t_*) vs. time. Another set of kinetic experiments were carried out to determine the kinetic order with respect to [*^i^*^Bu^SKA]. In these experiments, with the [B(C_6_F_5_)_3_]/[*^i^*^Bu^SKA] ratio was 1/0.5, 1/1, 1/2, 1/4, [MMBL]_0_ was fixed at 0.936 M and [B(C_6_F_5_)_3_]_0_ was fixed at 2.34 mM, where [*^i^*^Bu^SKA] = 1.17, 2.34, 4.68, 9.36 mM in 2.5 mL mixture solutions. The rest of the procedure was same as the described above.

### 3.13. Polymer Characterizations

Number-average molecular weight (*M*_n_) and molecular weight distributions (*Ð* = *M*_w_/*M*_n_) of polymers were measured by gel permeation chromatography (GPC) at 35 °C and a flow rate of 1 mL/min, with DMF (HPLC grade, containing 50 mmol/L LiBr) as an eluent on a Waters 1515 instrument (Milford, MA, USA) equipped with a Waters 4.6 × 30 mm guard column and three Waters WAT054466, WAT044226, WAT044223 columns (Polymer Laboratories, Milford, MA, USA; linear range of molecular weight = 500 to 4 × 10^6^). The instrument was calibrated with 10 PMMA standards, and chromatograms were processed with Waters Breeze 2 software (version: 6.01.2154.026; Milford, MA, USA). Tacticities of PMMA was measured by ^1^H-NMR in CDCl_3_, while ^13^C-NMR of P(M)MBL (co)polymers were recorded in DMSO-*d_6_*.

The isolated low-MW polymer samples were analyzed by matrix-assissted laser desorption/ionization time-of-flight mass spectroscopy (MALDI-TOF MS); the experiment was performed on a BrukerAutoflex speed TOF/TOF mass spectrometer (Karlsruhe, Baden-Württemberg, Germany) in linear, positive ion, reflector mode using a 25 KV accelerating voltage. A thin layer of 1% CF_3_COONa solution was first deposited on the target plate, followed by 0.6 μL of both sample and matrix (*trans*-2-[3-(4-tertbutylphenyl)-2-methyl-2-propylidene]malonitrile (DCTB), 20 mg/mL in THF). The spectra of the sample was externally calibrated using a peptide calibration mixture (4–6 peptides). The raw data were processed in the Bruker Daltonics FlexAnalysis 3.3.80.0 software (Karlsruhe, Baden-Württemberg, Germany).

## 4. Conclusions

In summary, FLPs based on bulky organo-LA, B(C_6_F_5_)_3_, and SKAs either preformed or in-situ generated from the hydrosilylation with R_3_SiH, were employed for GTP of polar vinyl monomers, including linear MMA as well as the cyclic renewable monomers MMBL and MBL. Although the polymerizations of MMA by both R_3_SiH/B(C_6_F_5_)_3_ and SKA/B(C_6_F_5_)_3_ systems are sluggish, SKA/B(C_6_F_5_)_3_ system promotes the living/controlled GTP of MMBL and MBL, achieving polymers with predicted *M*_n_ values and narrow *Đ* values. 

The formation of FLP was confirmed by in situ NMR reactions of SKA with B(C_6_F_5_)_3_. Detailed investigations into the characterization of key reaction intermediates, polymerization kinetics and polymer structures have led to a GTP polymerization mechanism, in which the reaction was initiated with the intermolecular Michael addition of the SKA enolate group to vinyl group of B(C_6_F_5_)_3_-activated monomer, while the silyl group is transferred to the carbonyl group of the monomer to generate the SKA analogues and release B(C_6_F_5_)_3_ which reenters the next catalytic cycle for the activation of incoming monomer. The living features of such *^i^*^Bu^SKA/B(C_6_F_5_)_3_FLP system enabled us to successfully synthesized well-defined diblock, triblock copolymer PMMBL-*b*-PMBL and PMMBL-*b*-PMBL-*b*-PMMBL with predicted *M*_n_ by using such ^iBu^SKA/B(C_6_F_5_)_3_ FLP system.

## Figures and Tables

**Table 1:** B(C_6_F_5_)_3_-catalyzed polymerization of (M)MBL *^a^.*

Run No.	Initiator (I)	Monomer (M)	[M]:[I]:[B] *^b^*	Time (h)	Conv. *^c^* (%)	*M*_n._ *^d^* (kg/mol)	*Ð*	I* *^e^* (%)
1	Me_2_EtSiH	MMBL	200:1:1	0.5	100	48.1	1.26	47
2	Me_2_PhSiH	MMBL	200:1:1	0.5	100	57.4	1.39	39
3	Me_2_ClSiH	MMBL	200:1:1	2	100	78.6	1.44	29
4	Et_3_SiH	MMBL	200:1:1	0.5	100	33	1.27	68
5	Ph_3_SiH	MMBL	200:1:1	6	100	105	1.61	21
6	*^i^*Bu_3_SiH	MMBL	200:1:1	24	100	345	1.51	7
7	^Me2Ph^SKA	MMBL	200:1:1	0.5	100	45.5	1.33	50
8	^Me2(EtO)^SKA	MMBL	200:1:1	0.5	100	41.5	1.37	54
9	^Me2Cl^SKA	MMBL	200:1:1	6	100	85.1	1.82	27
10	^Ph^SKA	MMBL	200:1:1	2	100	68.8	1.37	33
11	^Me^SKA	MMBL	200:1:1	1	100	31.9	1.08	71
12	^Et^SKA	MMBL	200:1:1	0.5	100	37	1.21	61
13	*^i^*^Bu^SKA	MMBL	200:1:1	1	100	32.2	1.09	70
14	*^i^*^Bu^SKA	MMBL	100:1:1	0.17	100	13.2	1.08	86
15	^Me^SKA	MBL	200:1:1	24	100	14.6 (58%) 98.0 (42%)	1.20, 1.80	-
16	^Me^SKA	MBL	100:1:1	0.5	100	12.7	1.12	78
17	*^i^*^Bu^SKA	MBL	200:1:1	24	100	23.4	1.95	84
18	*^i^*^Bu^SKA	MBL	100:1:1	0.5	100	13.4	1.05	74

*^a^* Carried out in 2.25 mL CH_2_Cl_2_ at room temperature, where [MMBL]_0_ or [MBL]_0_ = 0.936M. *^b^* [M] = [Monomer], [I] = [Initiator], and [B] = [B(C_6_F_5_)_3_]. *^c^* Monomer conversions measured by ^1^H NMR. *^d^ M*_n_ and *Ð* determined by GPC relative to PMMA standards in DMF. *^e^* Initiator efficiency I* (%) = *M*_n_(calcd)/*M*_n_(exptl) × 100, where *M*_n_(calcd) = [MW(MMBL)] × ([MMBL]_0_/[I]_0_) (conversion) + MW of chain-end groups.

**Table 2:** B(C_6_F_5_)_3_-catalyzed copolymerization of MMBL and MBL by *^i^*^Bu^SKA/B(C_6_F_5_)_3_ system *^a^.*

Run No.	M1/M2	Conv. *^b^*(%)	*M*_w_ *^c^* (kg/mol)	*Đ*
1 *^d^*	100 MMBL + 100 MBL	MBL:100 MMBL:100	28.3	1.03
2	100 MMBL/100 MBL	MBL:100 MMBL:100	25.7	1.07
3	100 MMBL/100 MBL/100 MMBL	MBL:100 MMBL:100	37.1	1.06

*^a^* Carried out in 2.25 mL CH_2_Cl_2_ at room temperature, where [MMBL]_0_ = [MBL]_0_ = 0.936 M and addition method: catalyst and monomer were premixed, followed by adding initiator to start the polymerization. *^b^* Monomer conversions measured by ^1^H-NMR. *^c^* *M*_n_ and MWD determined by GPC relative to PMMA standards in DMF. *^d^* MBL and MMBL was added at the same time.

## Supplementary Materials

Supplementary materials are available online.
