# Supplementary material for: Silyl Ketene Acetals/B(C6F5)3 Lewis Pair-Catalyzed Living Group Transfer Polymerization of Renewable Cyclic Acrylic Monomers
Source: Molecules. 2018 Mar 15;23(3):665. doi: 10.3390/molecules23030665 (PMC6017534; doi:10.3390/molecules23030665)
Supplement: Supplementary file 1 [file molecules-23-00665-s001.pdf]

Silyl Ketene Acetals/B(C_6F_5)$_3$Lewis Pair-Catalyzed Living Group Transfer Polymerization of Renewable Cyclic Acrylic Monomers

Lu Hu, Wuchao Zhao, Jianghua He,* and Yuetao Zhang*

State Key Laboratory of Supramolecular Structure and Materials, College of Chemistry, Jilin University, Changchun 130012,Jilin, China;hulu14@mails.jlu.edu.cn (L.H.); zhaowc16@mails.jlu.edu.cn (W.Z.)

* Correspondence: hjh2015@jlu.edu.cn (J.H.); ytzhang2009@jlu.edu.cn (Y.Z.); Tel.: +86-157-6432-7755(Y.Z.)

Received:30 January 2018; Accepted: 14 March 2018; Published: date

Table of Contens

1. Selected polymerization data ... S3

2. NMR spectrum of ^{Me2Cl}SKA ... S4

3. NMR spectrum of $Me_2EtOSiCl$ ... S5

4. NMR spectrum of $^{Me2(EtO)}$SKA ... S6

5. NMR spectrum of $B(C_6F_5)_3$·MMA ... S7

6. NMR spectrum of $B(C_6F_5)_3$·MMBL ... S8

7. NMR spectrum of the reaction of SKA with $B(C_6F_5)_3$ in 1:1 ratio ... S9

8. NMR spectrum of the reaction of SKA with $B(C_6F_5)_3$·MMA in 1:1 ratio ... S11

9. ^{13}C NMR spectrum of (co)polymers. ... S14

10. Plots of M_n and $Đ$ values of PMMBL samples vs $[MMBL]_0/[B(C_6F_5)_3]_0$ ratio ... S14

11. The GPC traces of PMMBL-*r*-PMBL ... S15

1. Selected polymerization data

Table S1. $B(C_6F_5)_3$-catalyzed MMA polymerization [a]

Run No.	Initiator (I)	[M]:[I]:[B][b]	Time (h)	Conv.[c] (%)	M_n[d] (Kg·mol^{-1})	Đ	I^*[e] (%)
1	Me_2PhSiH	200:1:1	24	4.46	n.d.	n.d.	n.d.
2	Me_2PhSiH	50:1:1	24	18.1	104	1.24	1
3[e]	Me_2PhSiH	200:1:1	24	12.9	296	1.55	1
4	Me_2EtSiH	200:1:1	24	5.67	n.d.	n.d.	n.d.
5	Me_2EtSiH	50:1:1	24	51.9	3	1.06	88
6[e]	Me_2EtSiH	200:1:1	24	12.1	428	1.33	0.6
7	Et_3SiH	200:1:1	24	6.24	n.d.	n.d.	n.d.
8	Et_3SiH	50:1:1	24	18.37	94	1.31	1
9[e]	Et_3SiH	200:1:1	24	9.24	305	1.55	0.6
10	Ph_3SiH	200:1:1	24	4.34	n.d.	n.d.	n.d.
11	Ph_3SiH	50:1:1	24	10.7	192	1.55	0.3
12[e]	Ph_3SiH	200:1:1	24	6.05	691	1.14	0.2
13	$^iBu_3SiH$	200:1:1	24	7.13	n.d.	n.d.	n.d.
14	$^iBu_3SiH$	50:1:1	24	13.9	234	1.53	0.3
15[e]	$^iBu_3SiH$	200:1:1	24	8.88	668	1.09	0.3
16	Me_2ClSiH	200:1:1	24	4.24	n.d.	n.d.	n.d.
17	Me_2ClSiH	50:1:1	24	11.3	101	1.26	0.6
18[e]	Me_2ClSiH	200:1:1	24	9.7	367	1.43	0.5
19	^{Me}SKA	200:1:1	24	5	n.d.	n.d.	n.d.
20	^{Me}SKA	50:1:1	24	47. 2	3.1	1.05	77
21[e]	^{Me}SKA	200:1:1	24	13.2	312	1.49	1
22	^{Et}SKA	200:1:1	24	5.59	n.d.	n.d.	n.d.
23	^{Et}SKA	50:1:1	24	18.2	106	1.25	1
24[e]	^{Et}SKA	200:1:1	24	11.2	358	1.4	0.6
25	^{iBu}SKA	200:1:1	24	6.36	n.d.	n.d.	n.d.
26	^{iBu}SKA	50:1:1	24	29.2	2.8	1.01	53
27[e]	^{iBu}SKA	200:1:1	24	69.4	705	1.08	0.2
28	^{Ph}SKA	200:1:1	24	4.59	n.d.	n.d.	n.d.
29	^{Ph}SKA	50:1:1	24	11.9	74.3	1.53	0.8
30[e]	^{Ph}SKA	200:1:1	24	12.2	377	1.35	0.7
31	$^{Me2Cl}SKA$	200:1:1	24	6.10	n.d.	n.d.	n.d.
32	$^{Me2Cl}SKA$	50:1:1	24	13.7	88.5	1.33	0.8
33[e]	$^{Me2Cl}SKA$	200:1:1	24	8.77	427	1.35	0.4
34	$^{Me2(EtO)}SKA$	200:1:1	24	23.5	145	1.25	3
35	$^{Me2(EtO)}SKA$	50:1:1	24	54.6	3.1	1.08	89
36[e]	$^{Me2(EtO)}SKA$	200:1:1	24	30.8	246	1.51	3
37	$^{Me2Ph}SKA$	200:1:1	24	4.26	n.d.	n.d.	n.d.
38	$^{Me2Ph}SKA$	50:1:1	24	14.8	78.8	1.48	1
39[e]	$^{Me2Ph}SKA$	200:1:1	24	9.79	315	1.48	0.6

[a] Carried out in 9 mL CH_2Cl_2 at room temperature, where $[MMA]_0 = 0.943$ M, n.d. = not determined. [b] [M] = [Monomer], [I] = [Initiator], and [B] = [$B(C_6F_5)_3$]. [c]Monomer conversions measured by ^{1}H NMR. [d]M_n and $Đ$ determined by GPC relative to PMMA standards in DMF. [e] Initiator efficiency (I^*)% = M_n(calcd)/M_n(exptl) × 100, where M_n(calcd) = [MW(MMA)]×($[MMA]_0/[I]_0$) (conversion) + MW of chain-end groups. [e]$[MMA]_0 = 3.77$ M.

2. NMR spectrum of ^{Me2Cl}SKA

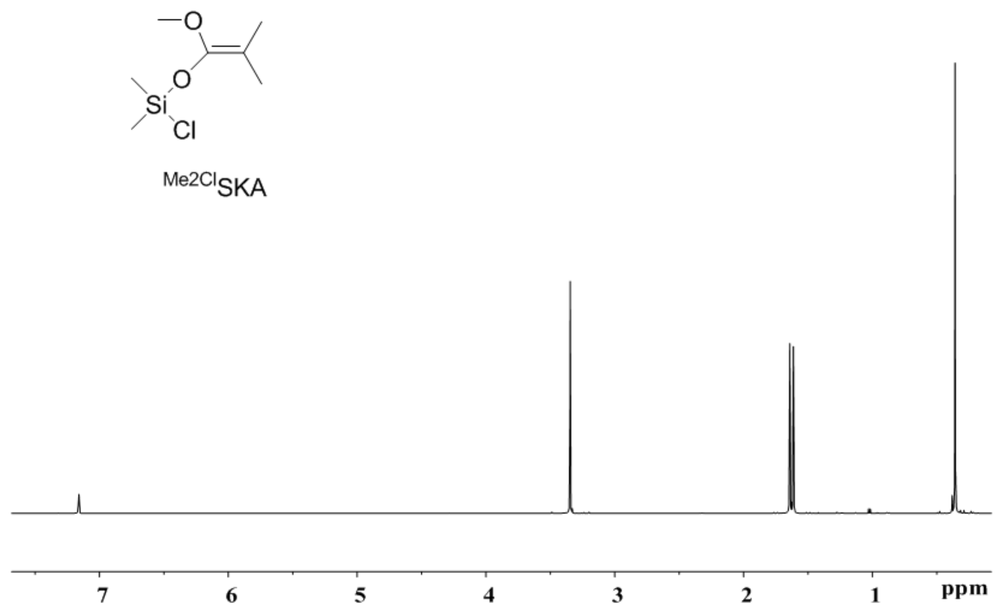

Figure S1. ^{1}H NMR spectrum (benzene-d_6, 500 MHz) of ^{Me2Cl}SKA.

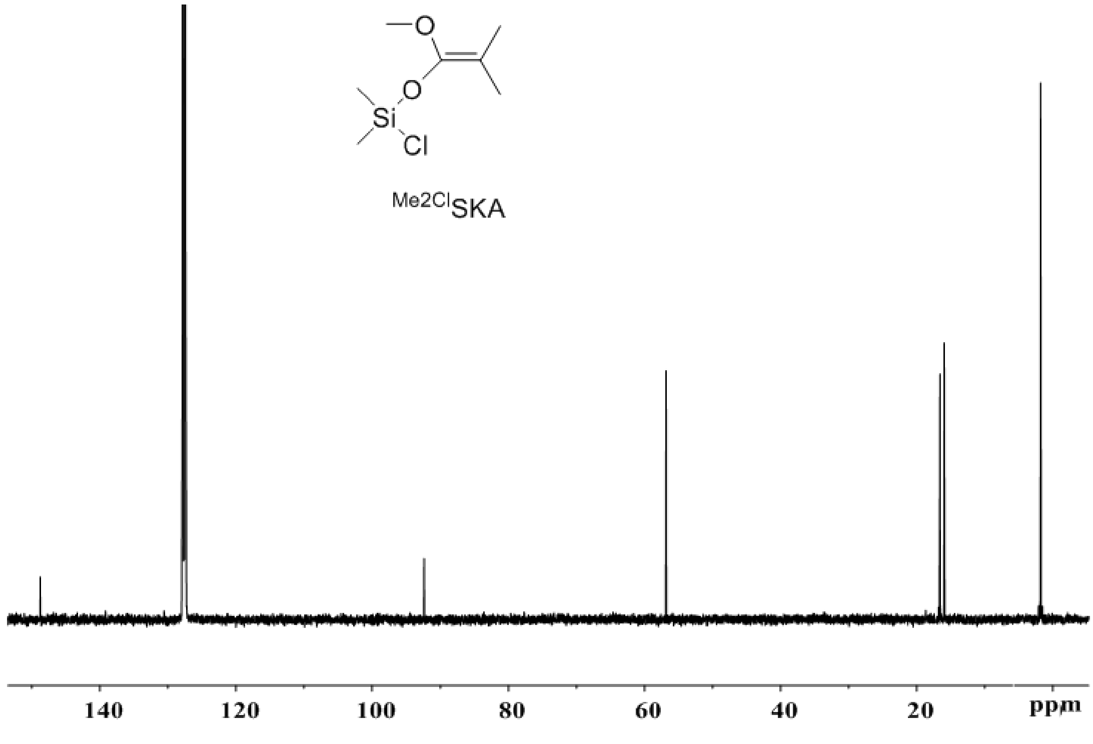

Figure S2. ^{13}C NMR spectrum (benzene-d_6, 126 MHz) of ^{Me2Cl}SKA.

3. NMR spectrum of $Me_2EtOSiCl$

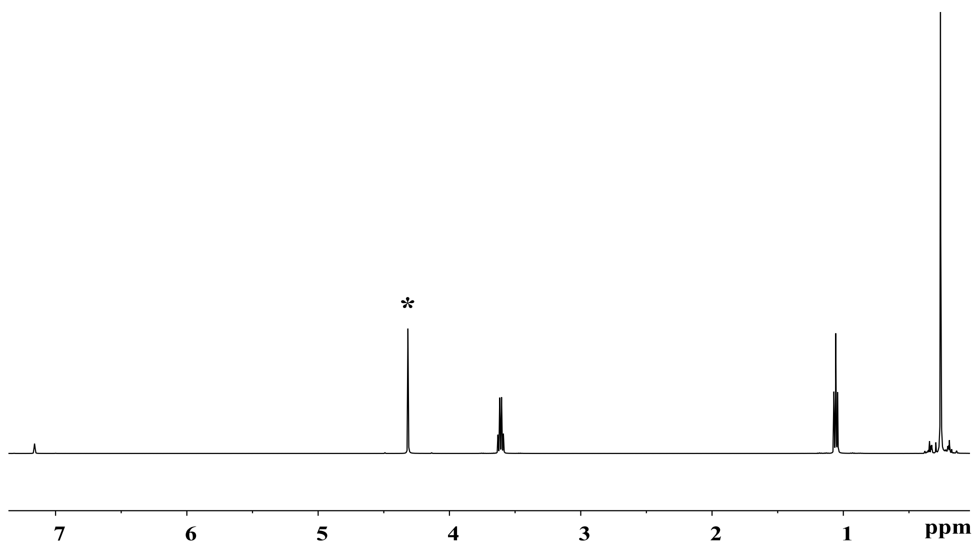

Figure S3. ^{1}H NMR spectrum of (benzene-d_6, 500 MHz) **$Me_2EtOSiCl$**.This spectrum also contain CH_2Cl_2 (peak marked with an *)

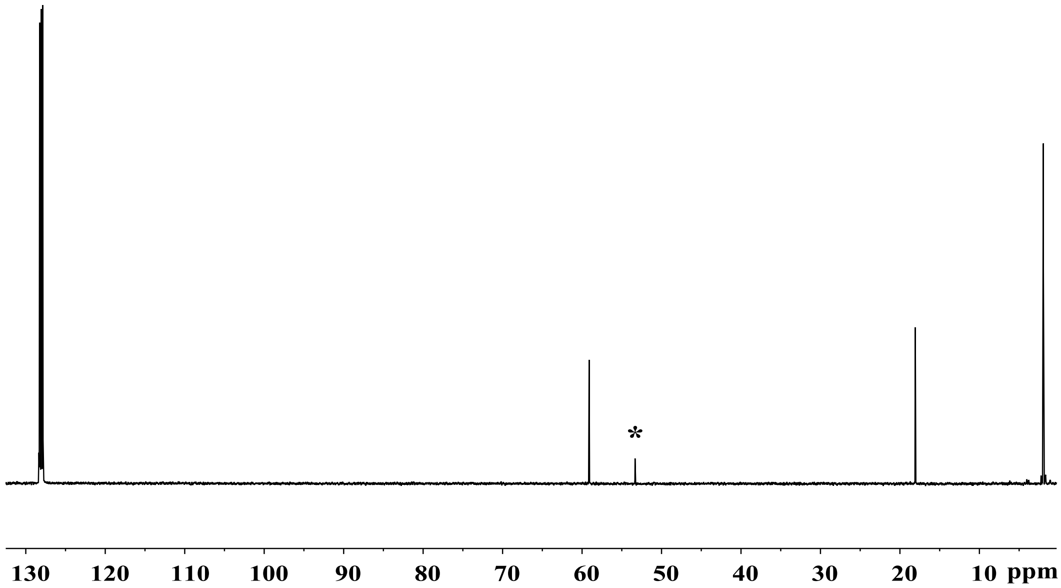

Figure S4. ^{13}C NMR spectrum (benzene-d_6, 126 MHz) of **$Me_2EtOSiCl$**. This spectrum also contain CH_2Cl_2 (peak marked with an *)

4. NMR spectrum of $^{Me2(EtO)}$SKA

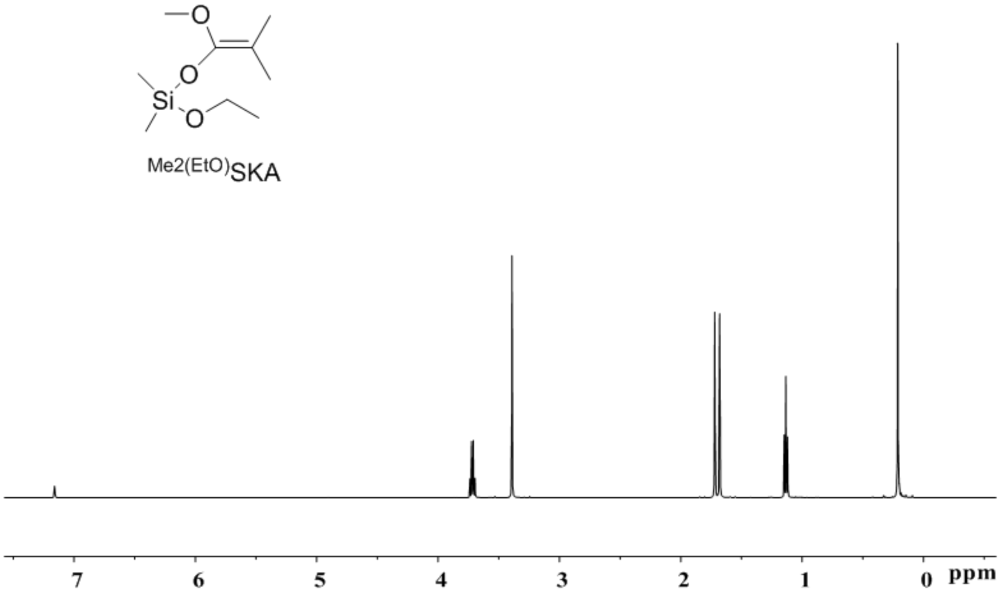

Figure S5. ^{1}H NMR spectrum (benzene-d_6, 500 MHz) of $^{Me2(EtO)}$SKA.

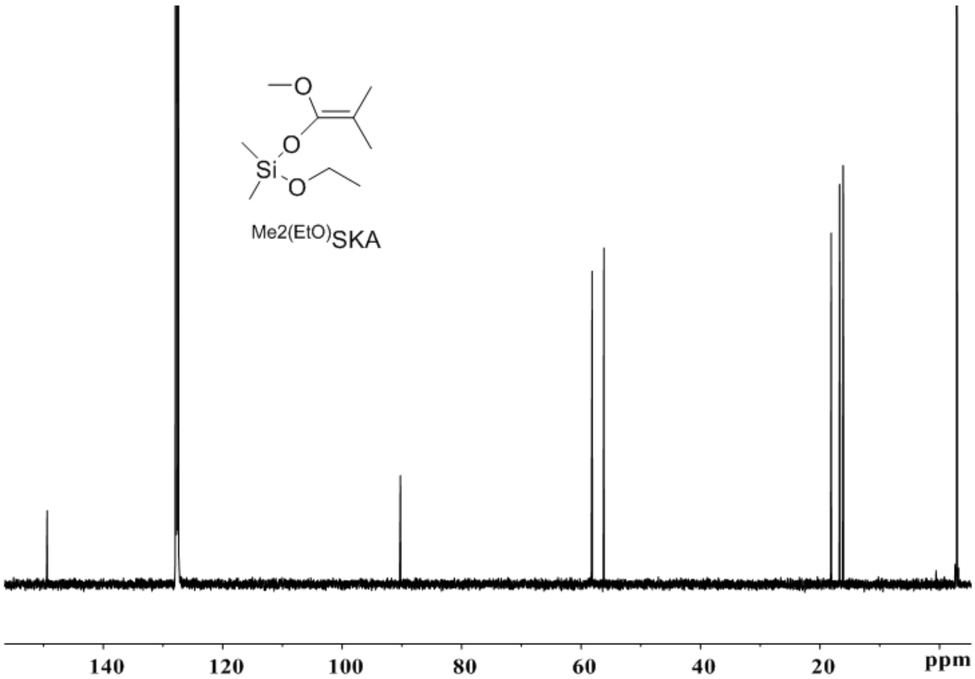

Figure S6. ^{13}C NMR spectrum (benzene-d_6, 126 MHz) of ^{Me2EtO}SKA

5. NMR spectrum of B(C_6F_5)$_3$·MMA

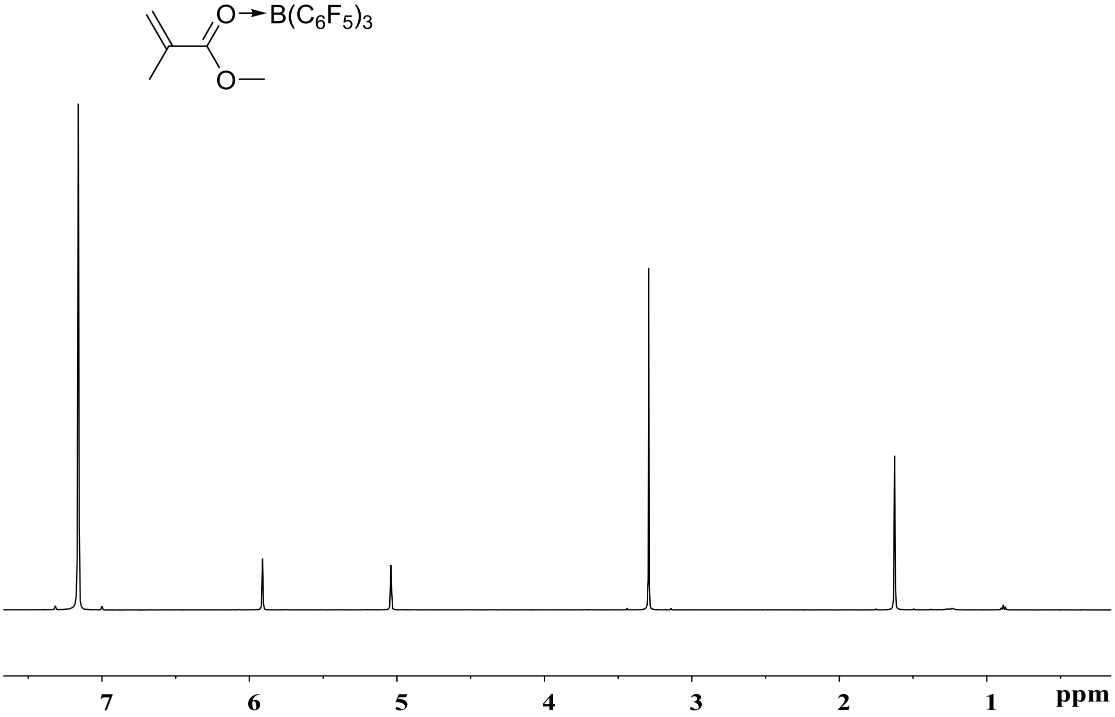

Figure S7. ^{1}H NMR spectrum (benzene-d_6, 500 MHz) of B(C_6F_5)$_3$·MMA.

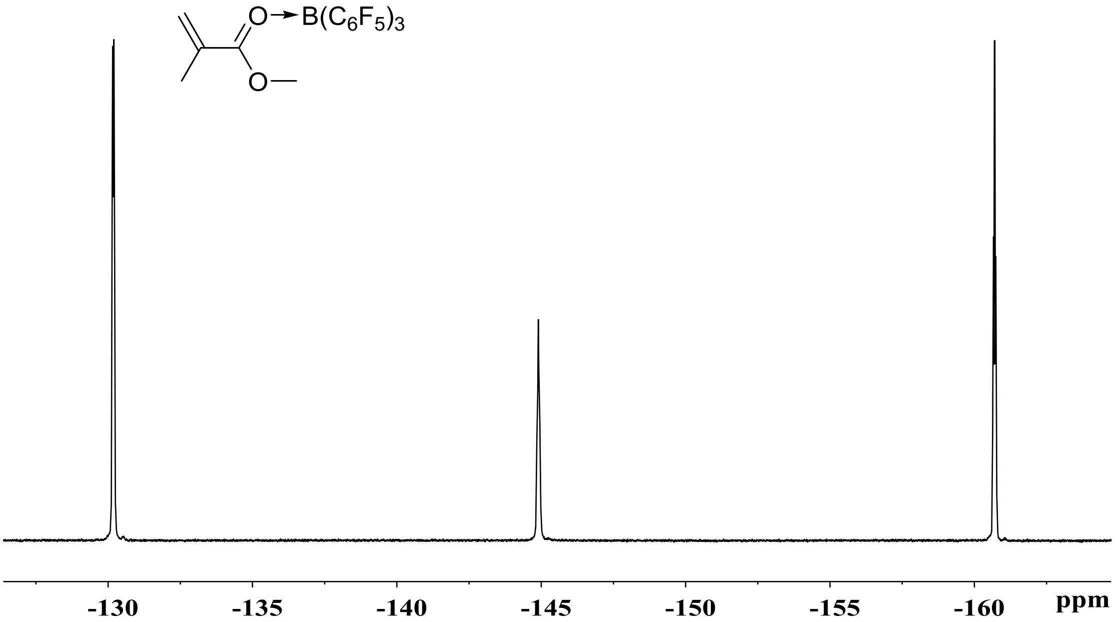

Figure S8. ^{19}F NMR spectrum (benzene-d_6, 471 MHz) of B(C_6F_5)$_3$·MMA.

6. NMR spectrum of $B(C_6F_5)_3$·MMBL

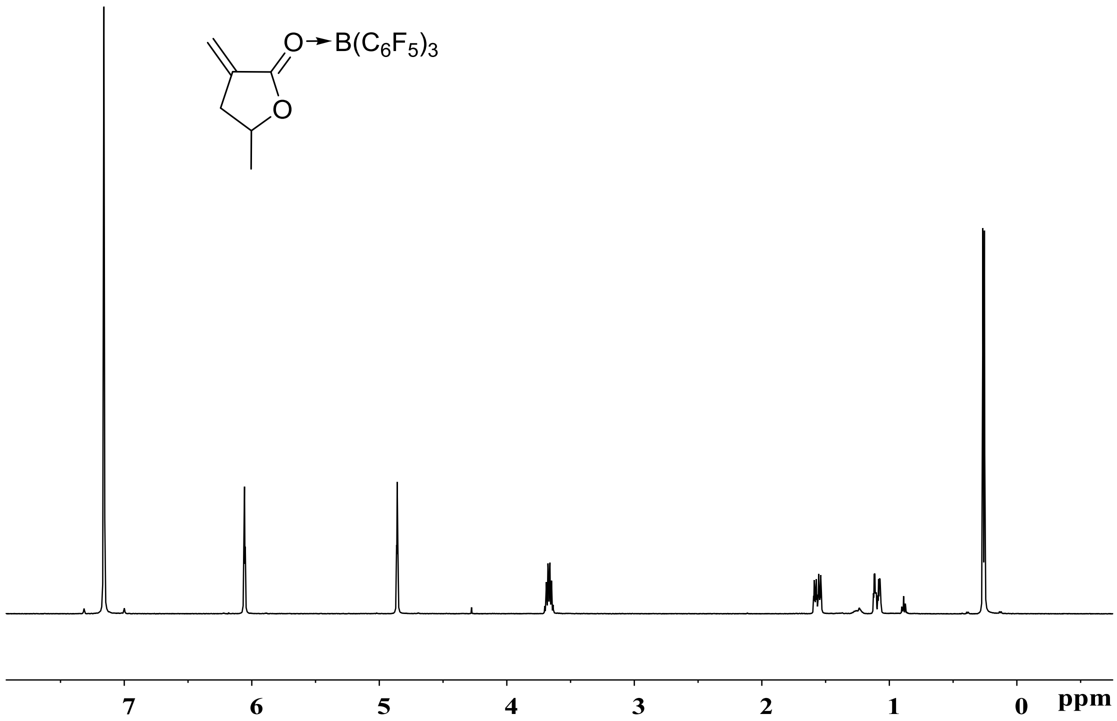

Figure S9. ^1H NMR spectrum (benzene-d_6, 500 MHz) of $B(C_6F_5)_3$·MMBL.

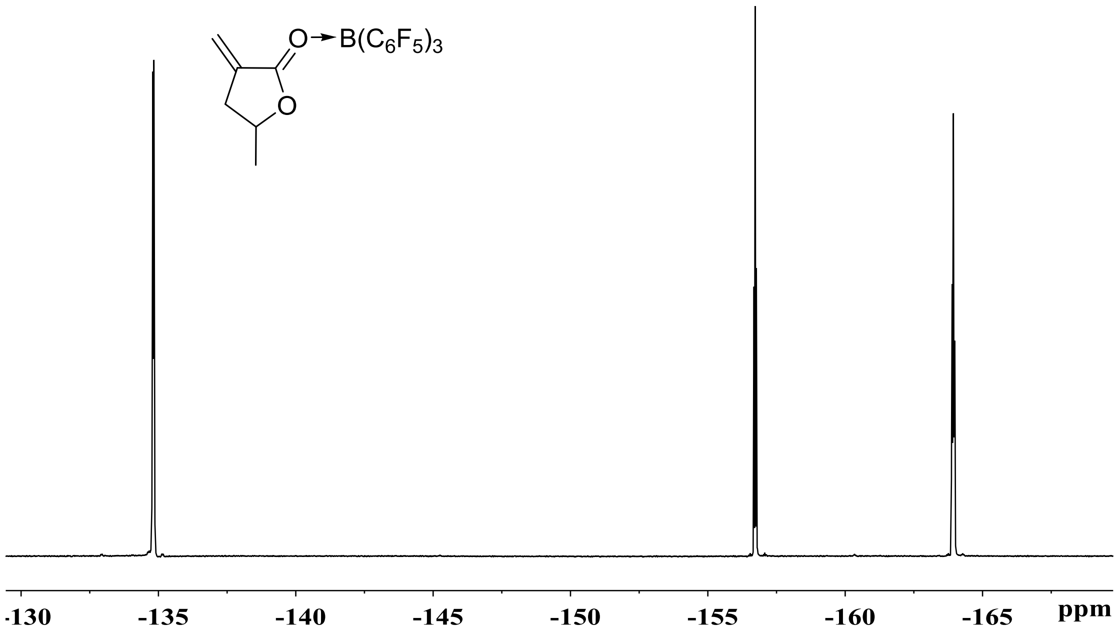

Figure S10. ^{19}F NMR spectrum (benzene-d_6, 471 MHz) of $B(C_6F_5)_3$·MMBL

7. NMR spectrum of the reaction of SKA with $B(C_6F_5)_3$ in 1:1 ratio

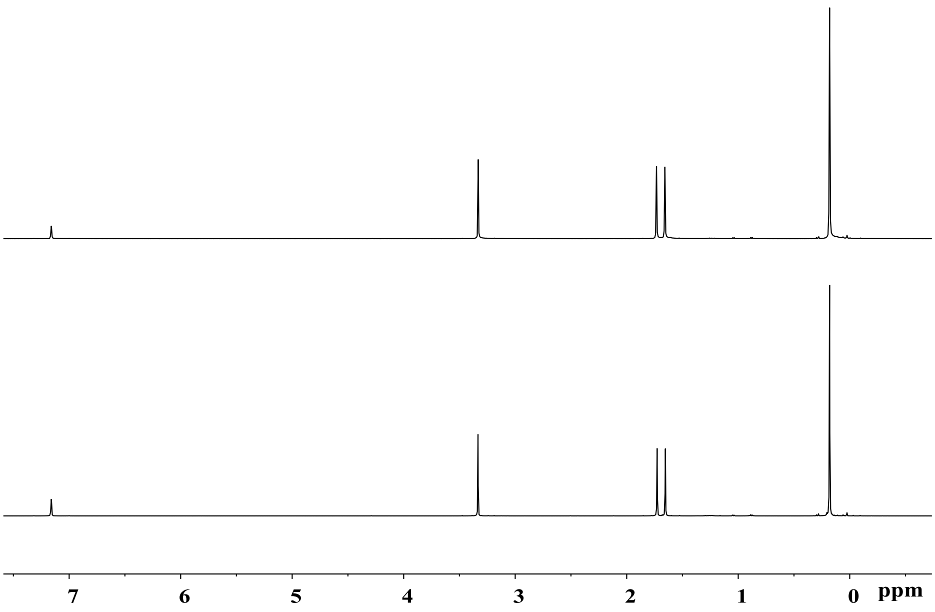

Figure S11. ^{1}H NMR spectrum (benzene-*d*$_6$,500 MHz) of ^{Me}SKA (Top) and the reaction with ^{Me}SKA/$B(C_6F_5)_3$ = 1:1ratio at RT (Bottom).

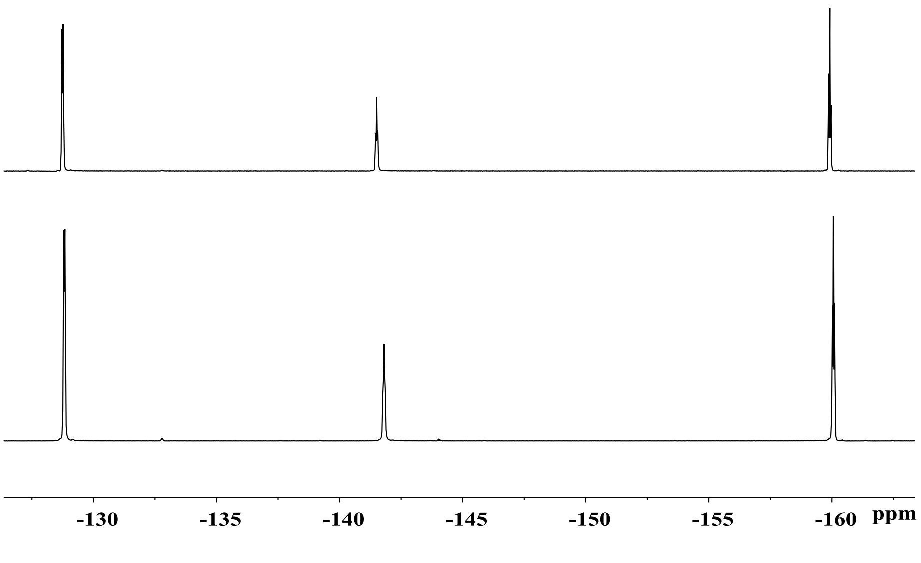

Figure S12. ^{19}F NMR spectrum (benzene-*d*$_6$, 471 MHz) of $B(C_6F_5)_3$ (Top) and the reaction with ^{Me}SKA/$B(C_6F_5)_3$ = 1:1ratio at RT (Bottom).

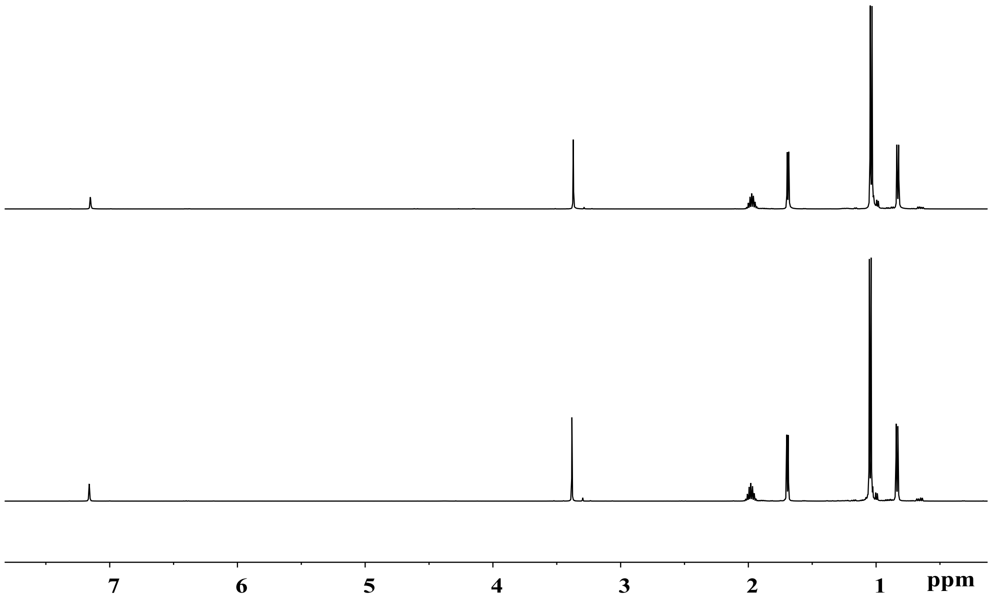

Figure S13.^{1}H NMR spectrum (benzene-d_6,500 MHz) of ^{iBu}SKA (Top) and the reaction with^{iBu}SKA/B(C_6F_5)$_3$ = 1:1ratio at RT (Bottom).

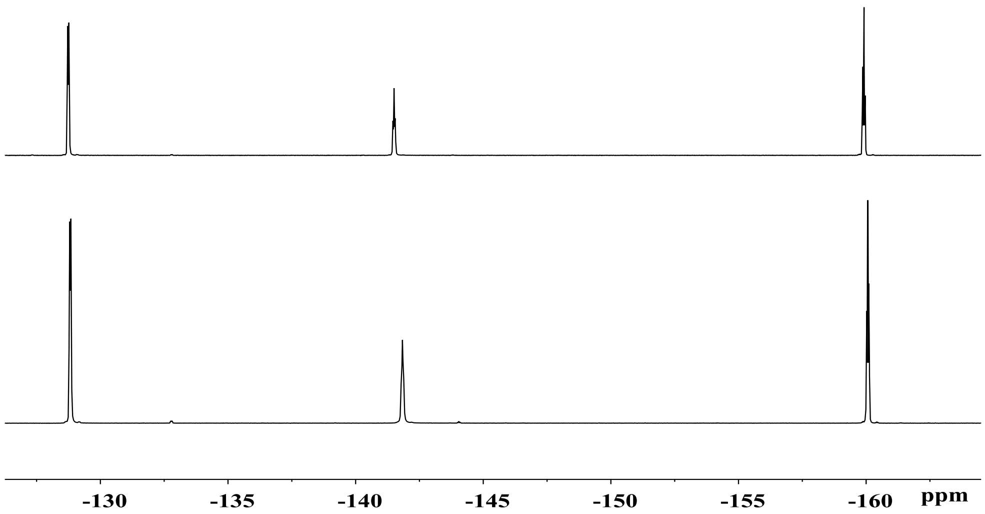

Figure S14. ^{19}F NMR spectrum (benzene-d_6, 471 MHz) of B(C_6F_5)$_3$ (Top) and the reaction with ^{iBu}SKA/B(C_6F_5)$_3$ = 1:1ratio at RT (Bottom).

8. NMR spectrum of the reaction of SKA with $B(C_6F_5)_3$·MMA in 1:1 ratio

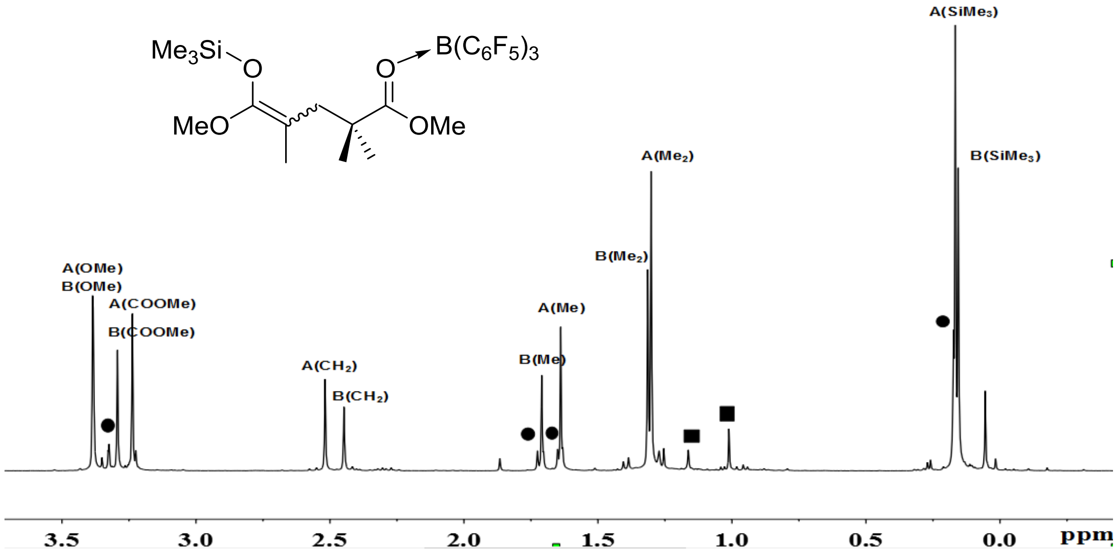

Figure S15. ^{1}H NMR spectrum (benzene-d_6, 500 MHz) of the reaction with ^{Me}SKA/$B(C_6F_5)_3$·MMA= 1:1ratio at RT. (major isomer **A** and minor isomer **B** in 3:2 ratio, the spectrum also contains a small amount of ^{Me}SKA (peaks marked with circle)

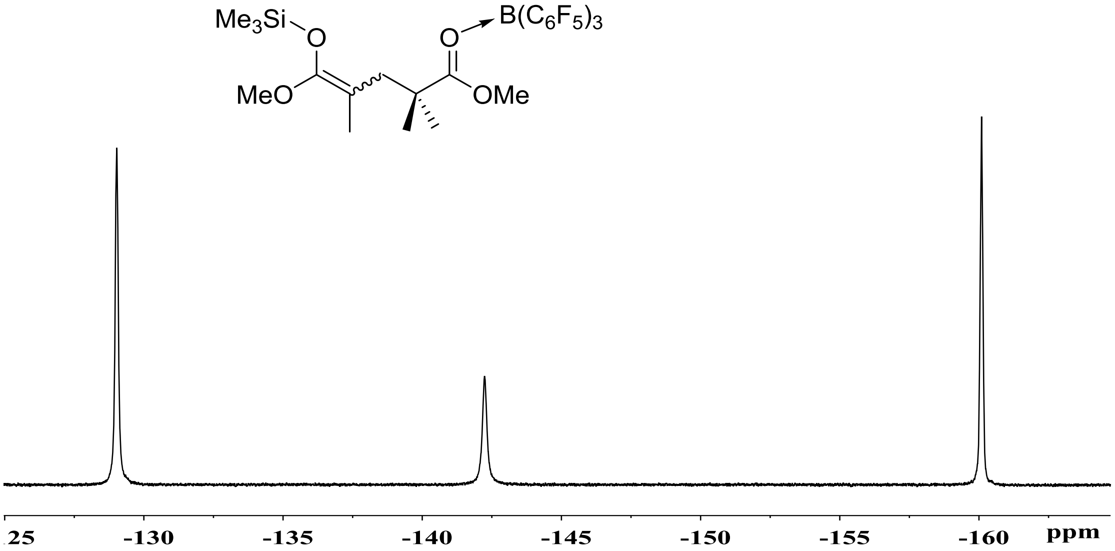

Figure S16. ^{19}F NMR spectrum (benzene-d_6, 471 MHz) of the reaction with ^{Me}SKA/$B(C_6F_5)_3$·MMA= 1:1 ratio at RT.

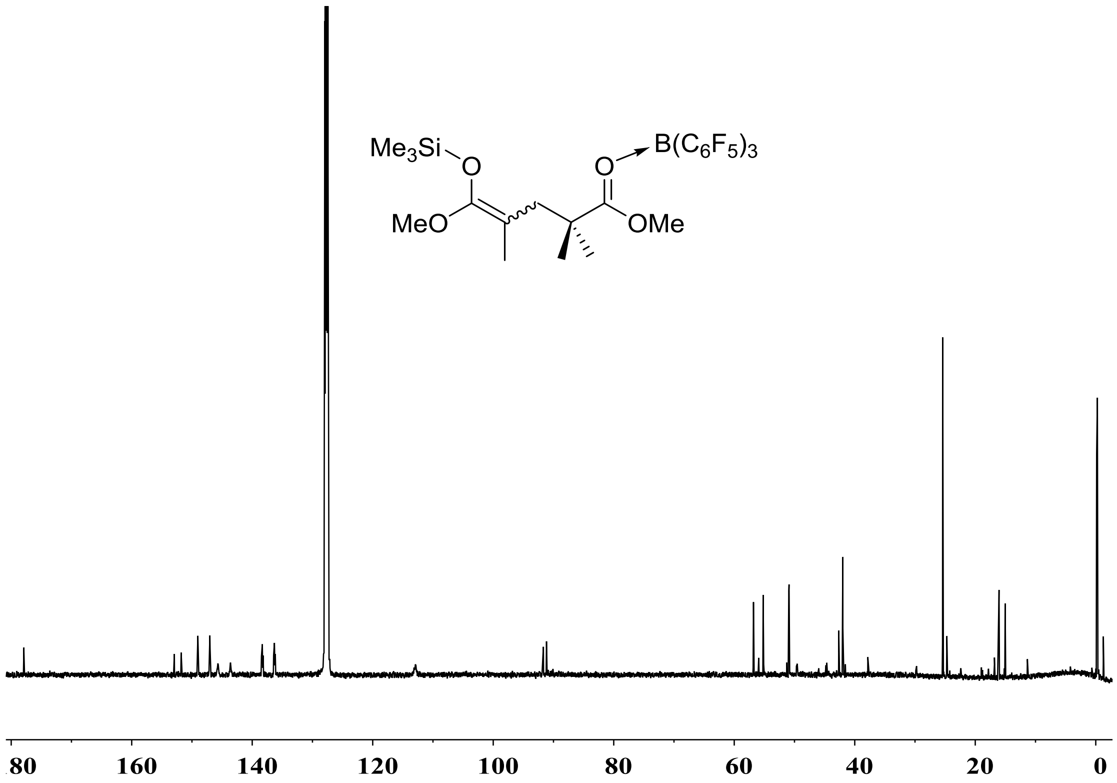

Figure S17. ^{13}C NMR spectrum (benzene-d_6, 126 MHz) of the reaction with ^{Me}SKA/ $B(C_6F_5)_3$·MMA= 1:1ratio at RT.

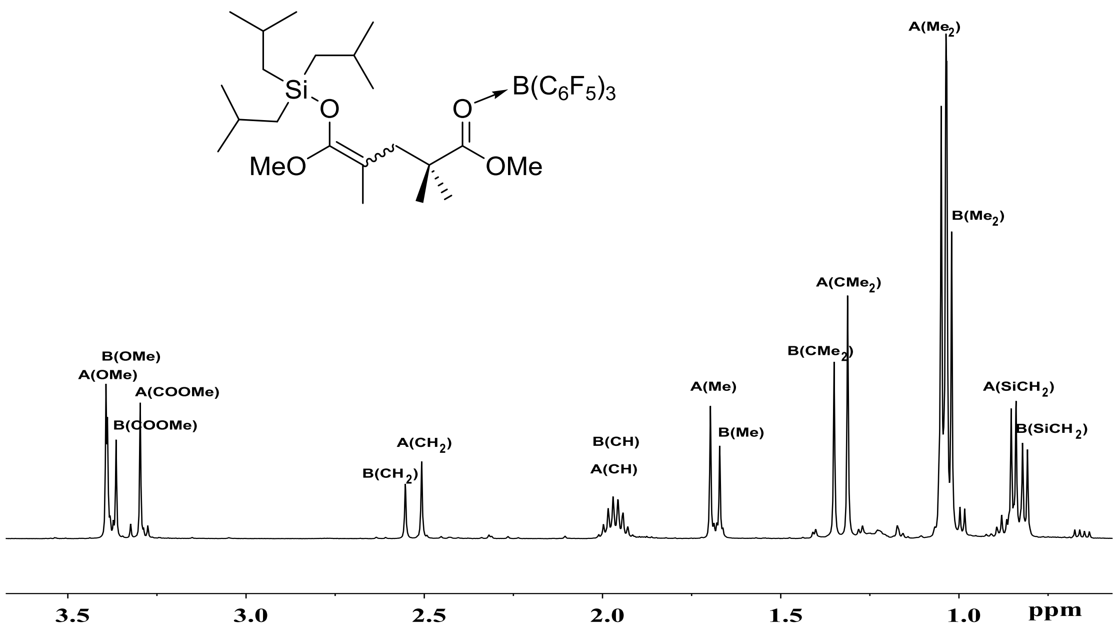

Figure S18. ^{1}H NMR spectrum (benzene-d_6, 500 MHz) of the reaction with ^{iBu}SKA/ $B(C_6F_5)_3$·MMA= 1:1 ratio at RT.

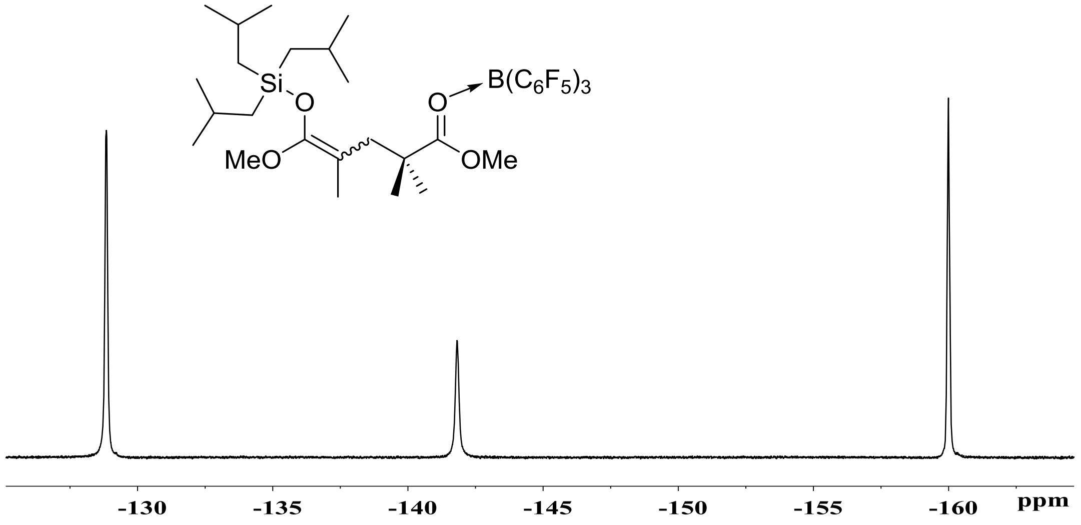

Figure S19.^{19}F NMR spectrum (benzene-d_6, 471 MHz) of the reaction with ^{iBu}SKA/ $B(C_6F_5)_3$·MMA= 1:1 ratio at RT.

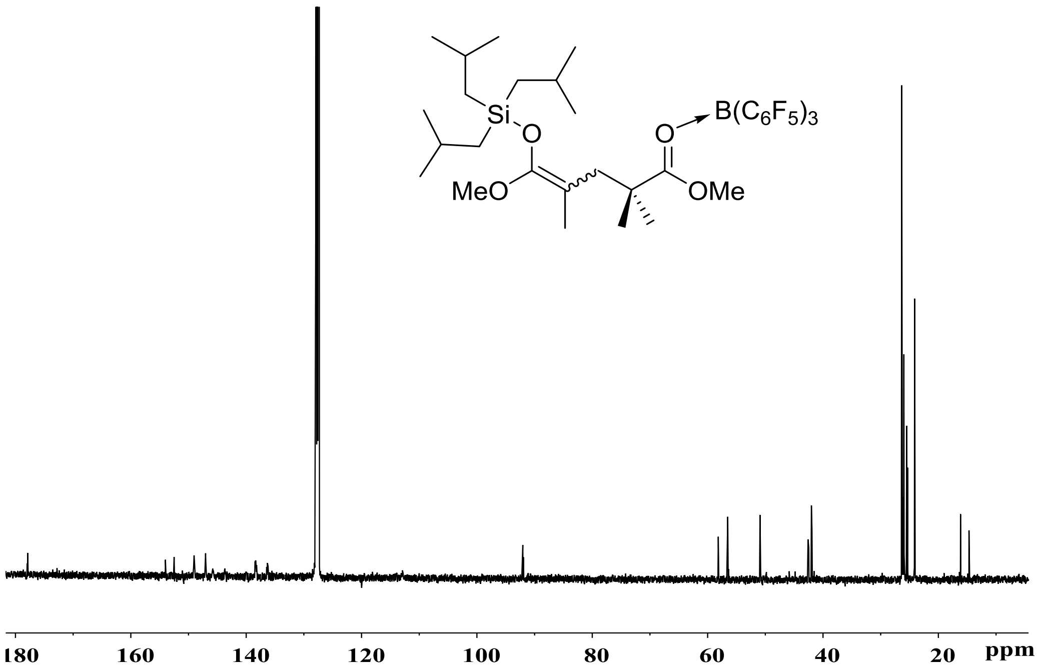

Figure S20.^{13}C NMR spectrum (benzene-d_6, 126 MHz) of the reaction with ^{iBu}SKA/ $B(C_6F_5)_3$·MMA= 1:1ratio at RT.

9. ^{13}C NMR spectrum of (co)polymers.

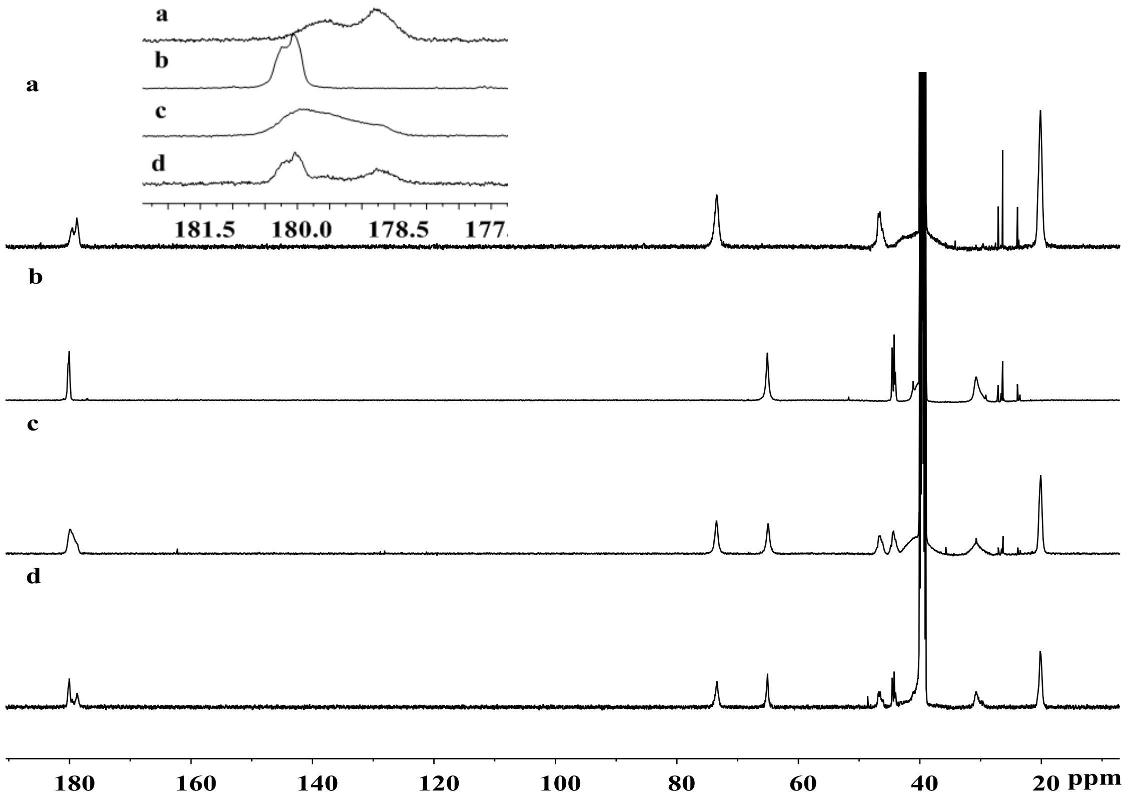

Figure S21. ^{13}C NMR spectrum (DMSO-d_6, 126 MHz) of (a) PMMBL, (b)PMBL, (c)random PMMBL-*r*-PMBL, (d) diblock PMMBL-*b*-PMBL and enlarged carbonyl signals (inset).

10. Plots of M_n and $Đ$ values of PMMBL samples vs [MMBL]$_0$/[B(C_6F_5)$_3$]$_0$ ratio

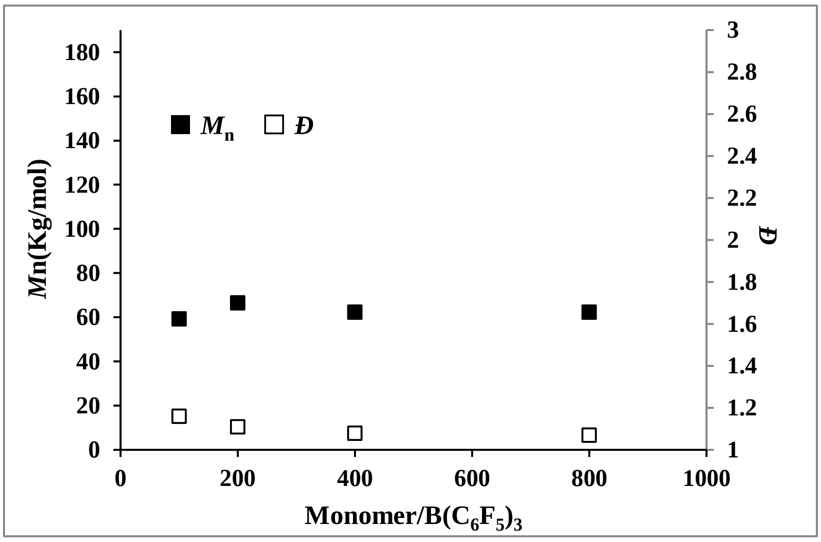

Figure S22. Plots of M_n and $Đ$ values of PMMBL samples vs [MMBL]$_0$/[B(C_6F_5)$_3$]$_0$ ratio at RT. Condition: [MMBL]$_0$/[^{iBu}SKA]$_0$/[B(C_6F_5)$_3$]$_0$ = 400:1:0.5, 400:1:1, 400:1:2, 400:1:4, R.T. [MMBL]$_0$ = 0.936M.

11. The GPC traces of PMMBL-*r*-PMBL

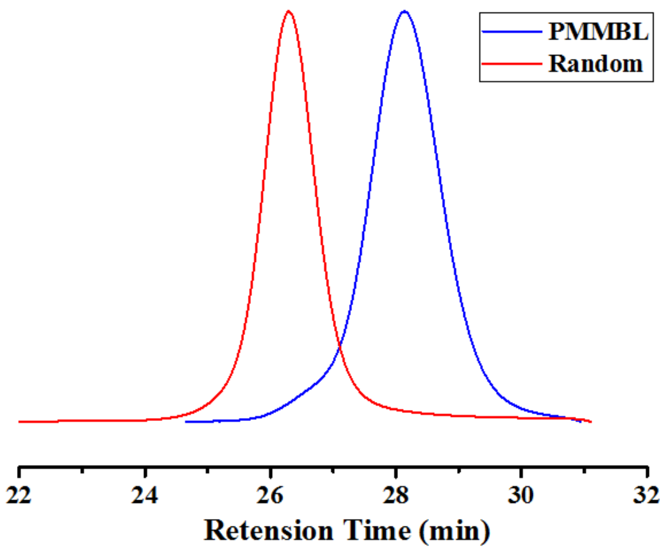

Figure S23. The GPC traces of homopolymer PMMBL (blue), and PMMBL-*r*-PMBL (red) produced by ^{iBu}SKA/$B(C_6F_5)_3$ in CH_2Cl_2 at RT.
